# The structural connectivity mapping of the intralaminar thalamic nuclei

**DOI:** 10.1038/s41598-023-38967-0

**Published:** 2023-07-24

**Authors:** Vinod Jangir Kumar, Klaus Scheffler, Wolfgang Grodd

**Affiliations:** 1 Max Planck Institute for Biological Cybernetics, Tübingen, Germany; 2 Department of Biomedical Magnetic Resonance, University Clinic Tübingen, Tübingen, Germany

**Keywords:** Neural circuits, Brain

## Abstract

The intralaminar nuclei of the thalamus play a pivotal role in awareness, conscious experience, arousal, sleep, vigilance, as well as in cognitive, sensory, and sexual processing. Nonetheless, in humans, little is known about the direct involvement of these nuclei in such multifaceted functions and their structural connections in the brain. Thus, examining the versatility of structural connectivity of the intralaminar nuclei with the rest of the brain seems reasonable. Herein, we attempt to show the direct structural connectivity of the intralaminar nuclei to diencephalic, mesencephalic, and cortical areas using probabilistic tracking of the diffusion data from the human connectome project. The intralaminar nuclei fiber distributions span a wide range of subcortical and cortical areas. Moreover, the central medial and parafascicular nucleus reveal similar connectivity to the temporal, visual, and frontal cortices with only slight variability. The central lateral nucleus displays a refined projection to the superior colliculus and fornix. The centromedian nucleus seems to be an essential component of the subcortical somatosensory system, as it mainly displays connectivity via the medial and superior cerebellar peduncle to the brainstem and the cerebellar lobules. The subparafascicular nucleus projects to the somatosensory processing areas. It is interesting to note that all intralaminar nuclei have connections to the brainstem. In brief, the structural connectivity of the intralaminar nuclei aligns with the structural core of various functional demands for arousal, emotion, cognition, sensory, vision, and motor processing. This study sheds light on our understanding of the structural connectivity of the intralaminar nuclei with cortical and subcortical structures, which is of great interest to a broader audience in clinical and neuroscience research.

## Introduction

**Figure 1:**
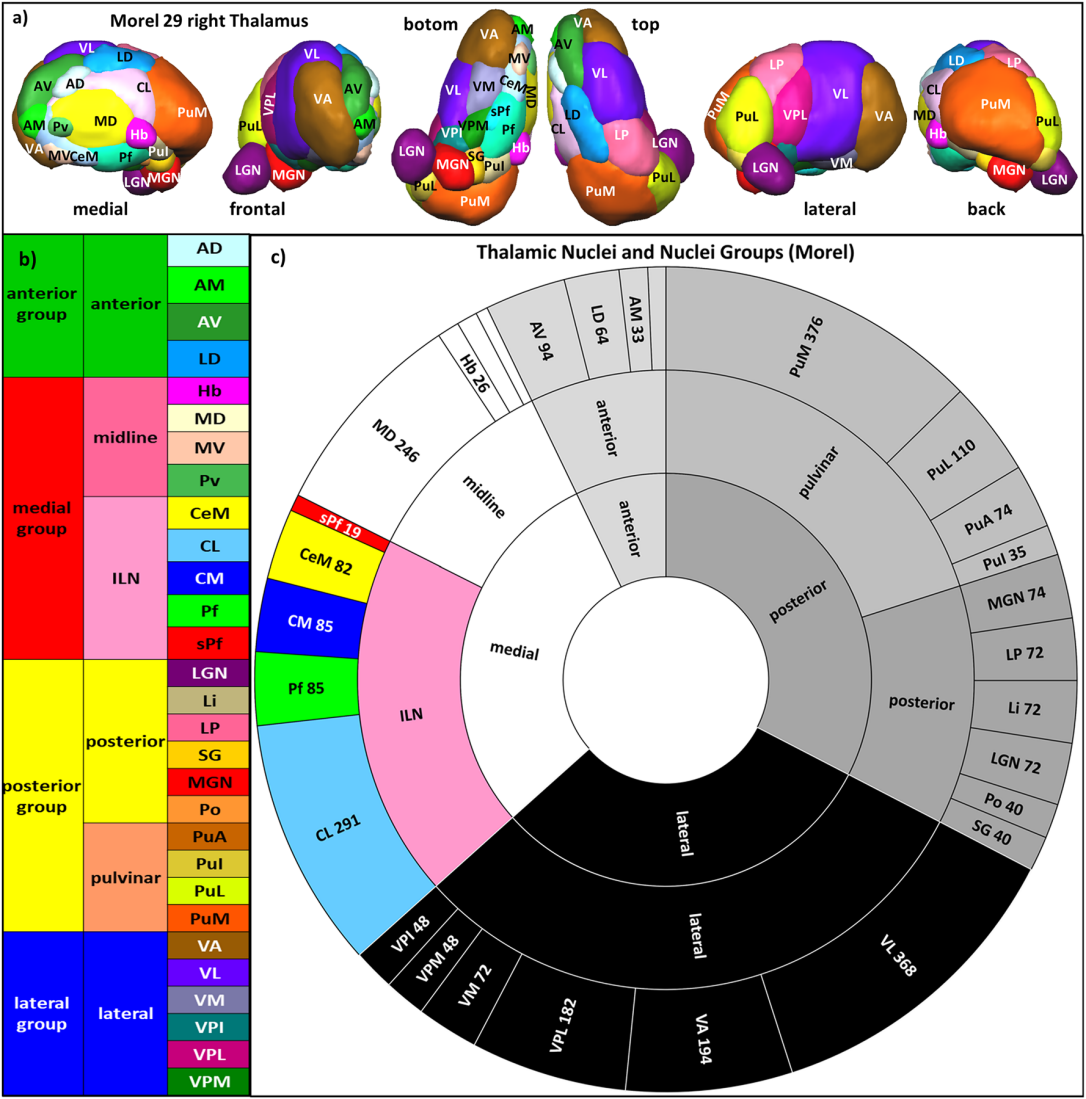
(**a**) Anatomy of the human thalamus: 3D Rendered views of 29 thalamic nuclei of the histological atlas of Morel with abbreviations (see Supplementary Table 1). (**b**) List of thalamic nuclei and nuclei groups. (**c**) Circle diagram of the distribution of nuclei and nuclei group with abbreviations and size in 2 mm^3^ voxels (according to the Atlas of Morel (2007) and Krauth et al. 2010).

**Figure 2:**
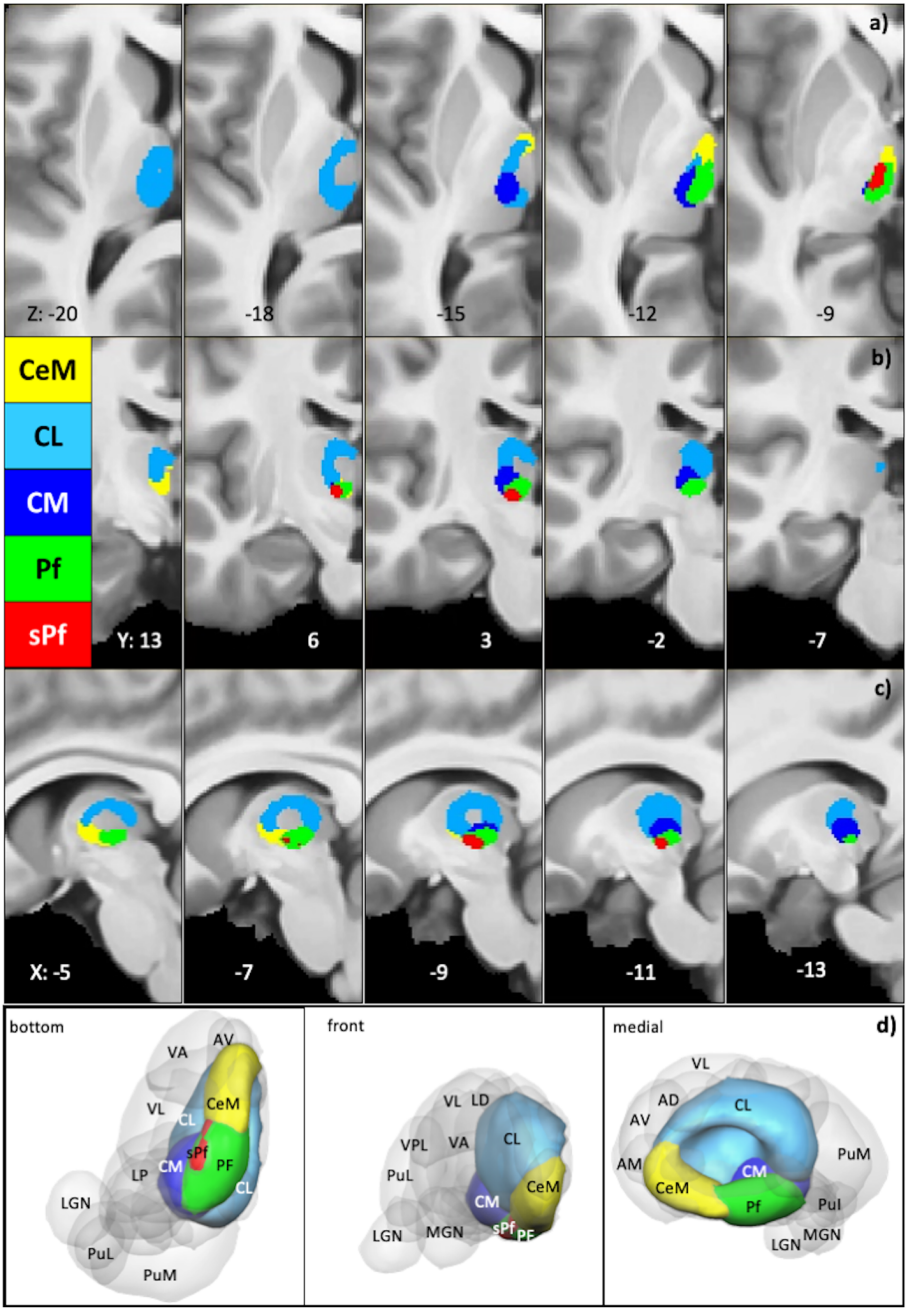
Intralaminar Nuclei Location Anatomy: location depiction of intralaminar nuclei within MRI 2D and 3D visualization of Intralaminar nuclei. (**a**–**c**) 2D maps show each intralaminar nuclei with its color code (depicted in the figure) on axial, coronal, and sagittal views. (**d**) 3D maps show each nucleus within the thalamus, with the whole brain perspectives from the bottom, front, and medial sides.

The thalamus is a mysterious and fascinating structure in the brain. It is intricately connected to various regions of the brain through projection fibers. The thalamus can be divided into four major groups of nuclei—the anterior, medial, lateral, and posterior groups—each with its specific function^1–5^ (Fig. 1, Supplementary Table 1). But within the medial group, there is a subgroup that stands out, the intralaminar nuclei (ILN). These nuclei are located within a unique and remarkable fiber pathway called the internal medullary lamina and are known to have a global influence on mental and cognitive function (Fig. 2). They diffusely project to different brain areas^6–9^, which enables them to control the transmission of information and the synchrony of the cortex^10^. The ILN also acts as a bridge between the brainstem and the cortex, facilitating rapid communication and processing of various functions such as awareness, conscious experience, perception, arousal, vigilance, sleep, visual, sensorimotor, attention, and sexual processing^11–20^. They are not only intriguing but also crucial in the understanding of disorders of consciousness, psychiatric conditions, and neurodegenerative diseases. They have even been identified as a target for deep brain neuromodulation in treating disorders of consciousness, highlighting their importance in brain functioning^21^.

Despite the ILN’s crucial role in multiple brain functions, a detailed understanding of each individual ILN and its structural connectivity with other brain regions remains elusive. While the ILN structural connectivity maps have been extensively determined in animal research^19^, they remain a mystery in humans. A handful of human studies have investigated the structural connectivity of the ILN as a group or complex^22,23^, but none have delved into the specific connectivity patterns of individual nuclei. This lack of information leaves many questions unanswered, and a deeper understanding of the structural connectivity of each ILN is essential to grasp their specific functions fully. Understanding structural connectivity can provide valuable insights, but we can only truly unravel the intricacies of the ILN's role in the brain by studying the individual nuclei. Therefore, it is essential to determine the structural connectivity patterns of the intralaminar nuclei in humans to understand further its role in various brain functions and the development as well as treatment of neurological and psychiatric disorders.

In the present study, we set out to unravel the mysterious ILN by delving into its structural connectivity patterns. We constructed detailed fiber connectivity maps for the five ILN nuclei using data from 730 healthy volunteers from the human connectome project (HCP)^24^. The study hypothesizes that the ILN communicates with various subcortical and cortical areas and constitutes different networks to facilitate diverse behavioral demands.

## Methods

### Data

The Young-healthy Human Connectome Project data (HCP, Principal Investigators: David Van Essen and Kamil Ugurbil; 1U54MH091657^25^; were used for this work. Using the HCP-900 release, we selected only subjects who completed the full imaging acquistion, resulting in 730 subjects (329 males and 401 females; age 22 to 37 years).

#### Data use of the Human Connectome Project

The study was performed in agreement with the WU-Minn HCP Consortium Open Access Data Use Terms of the HCP. The study used datasets from the HCP. We obtained HCP data use permission under open data use terms. Therefore, no further ethical approval was required. The HCP project (https://www.humanconnectomeproject.org/) is an open NIH initiative and got the required ethics approval for data acquisition and public distribution.

### MR Data Specification

#### Diffusion spectrum imaging (DSI)

The DSI data used a Spin-echo EPI sequence, TR: 5520 ms, TE: 89.5 ms, voxel size: 1.25 mm isotropic, multiband factor: 3, flip angle; 78 degrees; 111 slices, echo spacing: 0.78 ms, diffusion weighting consisted of 3 shells of b-values: 1000, 2000, and 3000 s/mm^2^, each with ~ 90 diffusion directions and 6 b = 0 acquisitions. The acquisition time was approximately 1 h^26^. For details, see^25,27^.

#### Structural imaging

T1w MPRAGE; voxel size 0.7 mm isotropic; 256 slices; Field of View (FOV) 224 × 224; TR 2400 ms; TE 2.14 ms; TI 1000 ms; Bandwidth 210 Hz/Px, IPAT 2; Flip Angle 8 degrees; Acquisition time 7:40 min: s.

#### Thalamus nuclei mask and Native space transformation

The digitized histological atlas of the human thalamus^28^ (Supplement Table 1, Fig. 1a) was aligned to the thalamus connectivity-based probability atlas space (Behrens et al., 2003; Johansen-Berg et al., 2005). The MNI-spaced nuclei were registered to each subject’s native diffusion space. The registration relies on linear and non-linear registration using FLIRT and FNIRT tools implemented in the FSL software (Supplementary Text). First, the linear registration from the non-diffusion to T1 volume and T1 to MNI space was computed in the registration procedure. In the next step, the non-linear registration was performed. In the third step, the inverse of the MNI to the non-diffusion space was calculated to register the nuclei into the native subject space^29,30^. The nuclei transformation allowed further diffusion calculations into the subject native space while maintaining high data quality and reducing registration interpolation errors^31^.

#### Diffusion-fit

The preprocessing included distortion and motion correction within the HCP pipeline^32–34^. The diffusion fit was performed using the FSL DTIFIT. The diffusion fit yielded color-coded FA maps in each subject. The visual inspection of each subject's FA map determined the quality of the diffusion fit.

#### Diffusion reconstruction

The reconstruction used a multishell model (three fibers per voxel, rician noise)^35^. The default noise was rician noise. Each subject's diffusion reconstruction was parallelized using sun-grid-engine (fsl_sub). The whole-brain multishell reconstruction required similar parameters for each subject.  

#### Probabilistic tractography

The probabilistic tractography was applied using FSL-*probtrackx*^36^*.* The probability diffusion algorithm repetitively samples from the distributions of voxel-wise principal diffusion directions by computing each time a streamline through the local samples to generate a probabilistic streamline or a sample from the distribution on the location of the true streamline. FMRIB's Diffusion Toolbox (FDT) builds up the histogram of the posterior distribution on the streamline location or the connectivity distribution^36^.

The probtrackx parameters included curvature threshold 80° (0.2), sample number 5000, step length 0.5, and a maximum number of steps: 2000. In the direct diffusion tractography, all streamlines passing through other nuclei were excluded from depicting only direct connections to the rest of the brain. The resulting tractograms were normalized by dividing them by the waytotal and multiplying them by 100.

### Native-subject-space Tractogram registration to MNI Space

The registration of the native-subject-spaced tracts to the MNI space relies on a combination of linear and non-linear registration steps (Supplementary Text). For each subject, a non-diffusion map to the structural T1 and T1 to 1 mm MNI brain was registered using the flirt linear registration method implemented in the FSL. The non-linear registration uses the output parameters from the linear registration and performs finer alignment to the MNI space using the fsl-fnirt method implemented in the FSL. Furthermore, using the above-generated non-linear and linear registrations, the fsl-applywarp tool was used to register the native-subject-spaced tractography maps to the MNI brain.

#### Group fixed effect analysis

The non-diffusion volume of each subject was coregistered to MNI brain space using a combination of linear and non-linear transformations described above. The resulting transformation matrices were then applied to the native-tractograms to align to the MNI space. The aligned tractograms from all subjects depicted the group fixed effect maps.

#### Visualization

**Figure 3:**
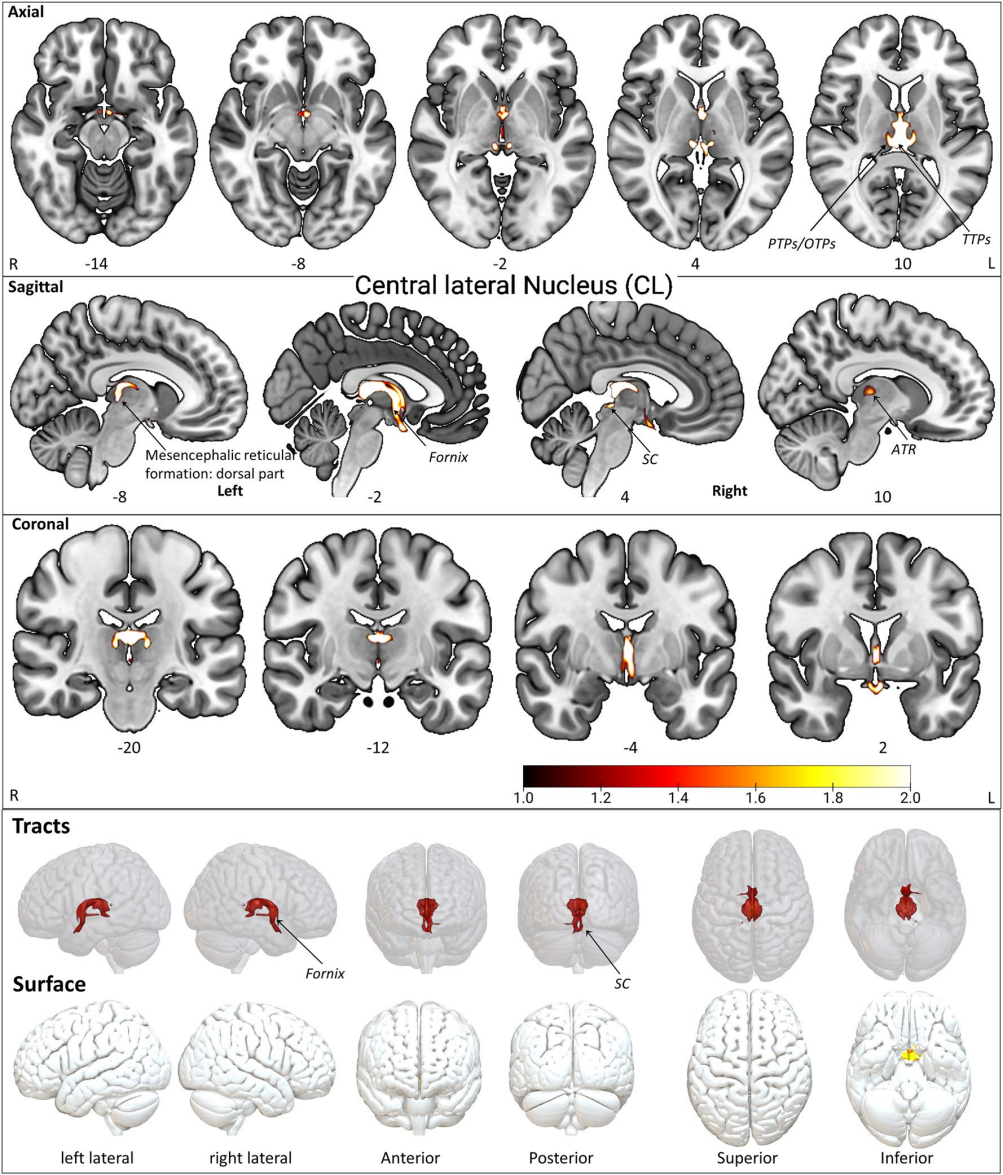
The CL tract fixed effect maps in axial, coronal, sagittal, 3D rendered, and surface views: *Connectivity:* The CL projections consist of SC, mesencephalic reticular formation (Dorsal part), and fornix. *PTPs* prefrontal thalamic projection site in oxford thalamus atlas, *TTPs* temporal thalamic projection in oxford thalamus atlas, *ATR* anterior thalamic radiation, *BS* brainstem, *SC* superior colliculus (Superior dorsal portion of the tectum in the midbrain). The detailed anatomical assignments are given in Supplementary Table 2.

**Figure 4:**
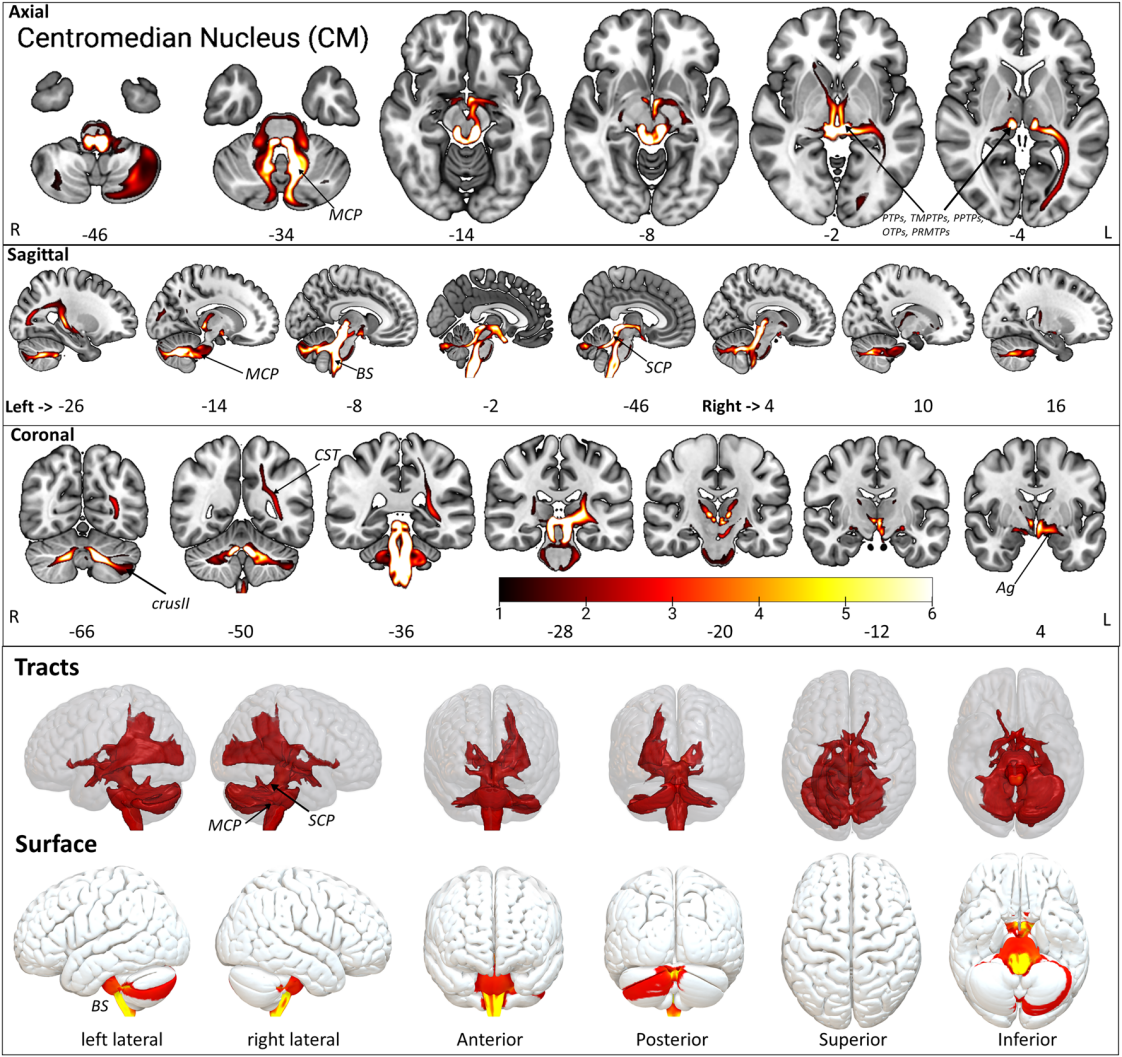
The CM tract fixed effect maps in axial, coronal, sagittal, 3D rendered, and surface views: *Connectivity:* The CM displayed connections to PMTPs, STPs, PTPs, MCP, SCP, cerebellar lobules (I-IV, V, VIIb, IX, CrusI, CrusII), BS, CST, Pd, and Ag. *PMTPs* premotor thalamic projection site in oxford thalamus atlas, *STPs* sensory thalamic projection site in oxford thalamus atlas, *PTPs* prefrontal thalamic projection site in oxford thalamus atlas, *MCP* medial cerebellar peduncle, *SCP* superior cerebellar peduncle, *BS* brainstem, *CST* pre-corticospinal tract site, *Pd* pallidum, *Ag* amygdala superficial group. The detailed anatomical assignments are given in Supplementary Table 3.

**Figure 5:**
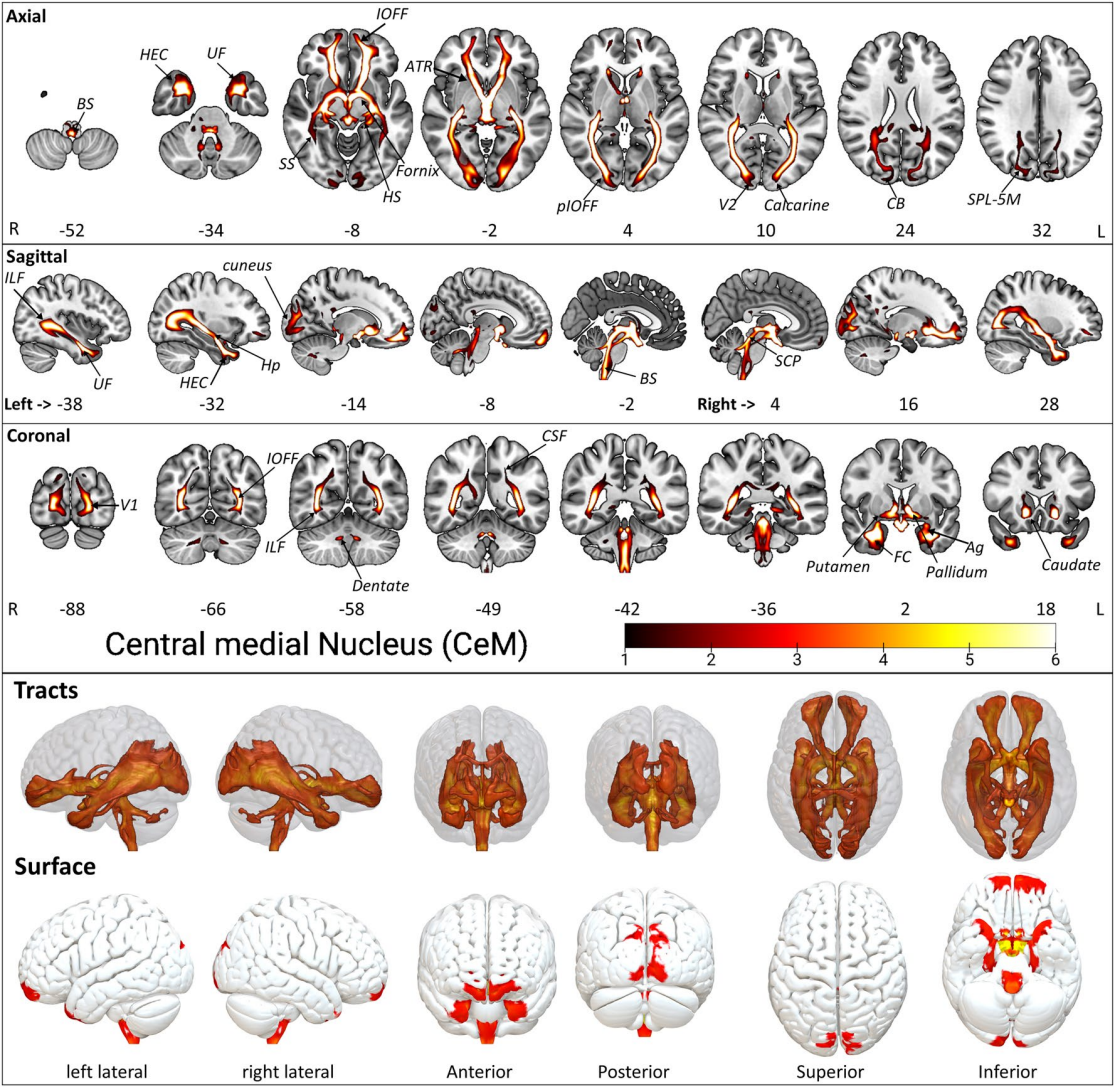
The CeM tract fixed effect maps in axial, coronal, sagittal, 3D rendered and surface views: *Connectivity:* The CeM projections consist of SCP, ICP, IOFF, aIOFFf, pIOFFf, MCP, CPCF, CSF, ILF, BS, HEC, UF, SPL-5M, CB, PTPs, SS, MGB, fornix, HS, Ag, OR, and the FC. *PTPs* prefrontal thalamic projection site in oxford thalamus atlas*, SCP* superior cerebellar peduncle, *ICP* inferior cerebellar peduncle, *IOFF* inferior occipito frontal fasciculus, *aIOFFf* anterior inferior occipito frontal fasciculus fragment, *pIOFFf* posterior inferior occipito frontal fasciculus fragment, *MCP* medial cerebellar peduncle, *CPCF* cortico ponto cerebellar fibers, *CSF* cortico spinal fibers, *ILF* inferior longitudinal fasciculus, *HEC* hippocampus entorhinal cortex, *BS* brainstem, *UF* uncinate fasciculus, *SPL-5M* superior parietal lobule 5M, *CB* callosal body, *SS* sagittal stratum (includes ILF & IFOF), *MGB* medial geniculate body, *HS* hippocampus subiculum, *Ag* amygdala laterobasal group, *OR* optic radiation, *FC* fusiform cortex. The detailed anatomical assignments are given in Supplementary Table 4.

**Figure 6:**
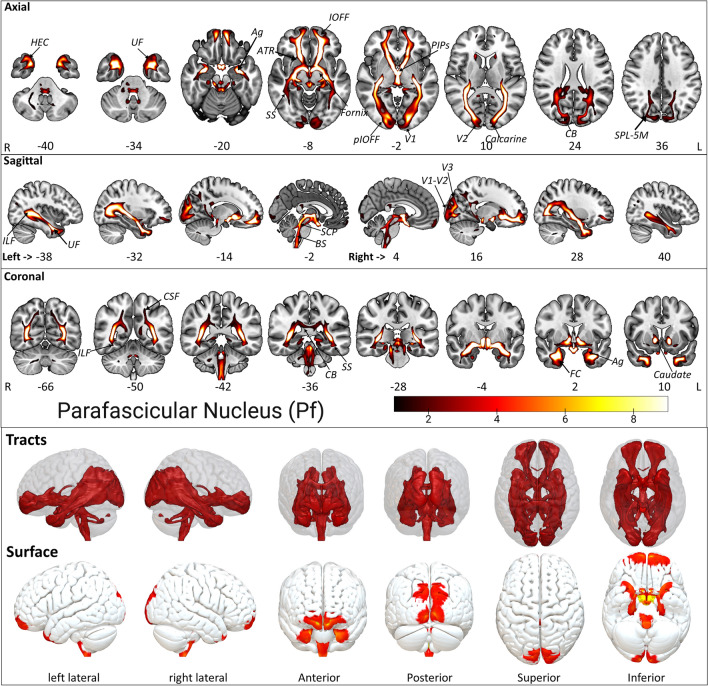
The Pf tract fixed effect maps in axial, coronal, sagittal, 3D rendered, and surface views: *Connectivity:* The Pf projections are similar to the CeM projections except for a slightly dominated connectivity distribution into the Lateral occipital cortex, precuneus, and splenium of the corpus callosum. In addition, the intrathalamic connections to the PTPs don't exist in Pf, unlike CeM. *PTPs* prefrontal thalamic projection site in oxford thalamus atlas*, SCP* superior cerebellar peduncle, *ICP* inferior cerebellar peduncle, *IOFF* inferior occipito frontal fasciculus, *aIOFFf* anterior inferior occipito frontal fasciculus fragment, *pIOFFf* posterior inferior occipito frontal fasciculus fragment, *MCP* medial cerebellar peduncle, *CPCF* cortico ponto cerebellar fibers, *CSF* cortico spinal fibers, *ILF* inferior longitudinal fasciculus, *HEC* hippocampus entorhinal cortex, *BS* brainstem, *UF* uncinate fasciculus, *SPL-5M* superior parietal lobule 5M, *CB* callosal body, *SS* sagittal stratum (includes ILF & IFOF), *MGB* medial geniculate body, *HS* hippocampus subiculum, *Ag* amygdala laterobasal group, *OR* optic radiation, *FC* fusiform cortex. The detailed anatomical assignments are given in Supplementary Table 5.

**Figure 7:**
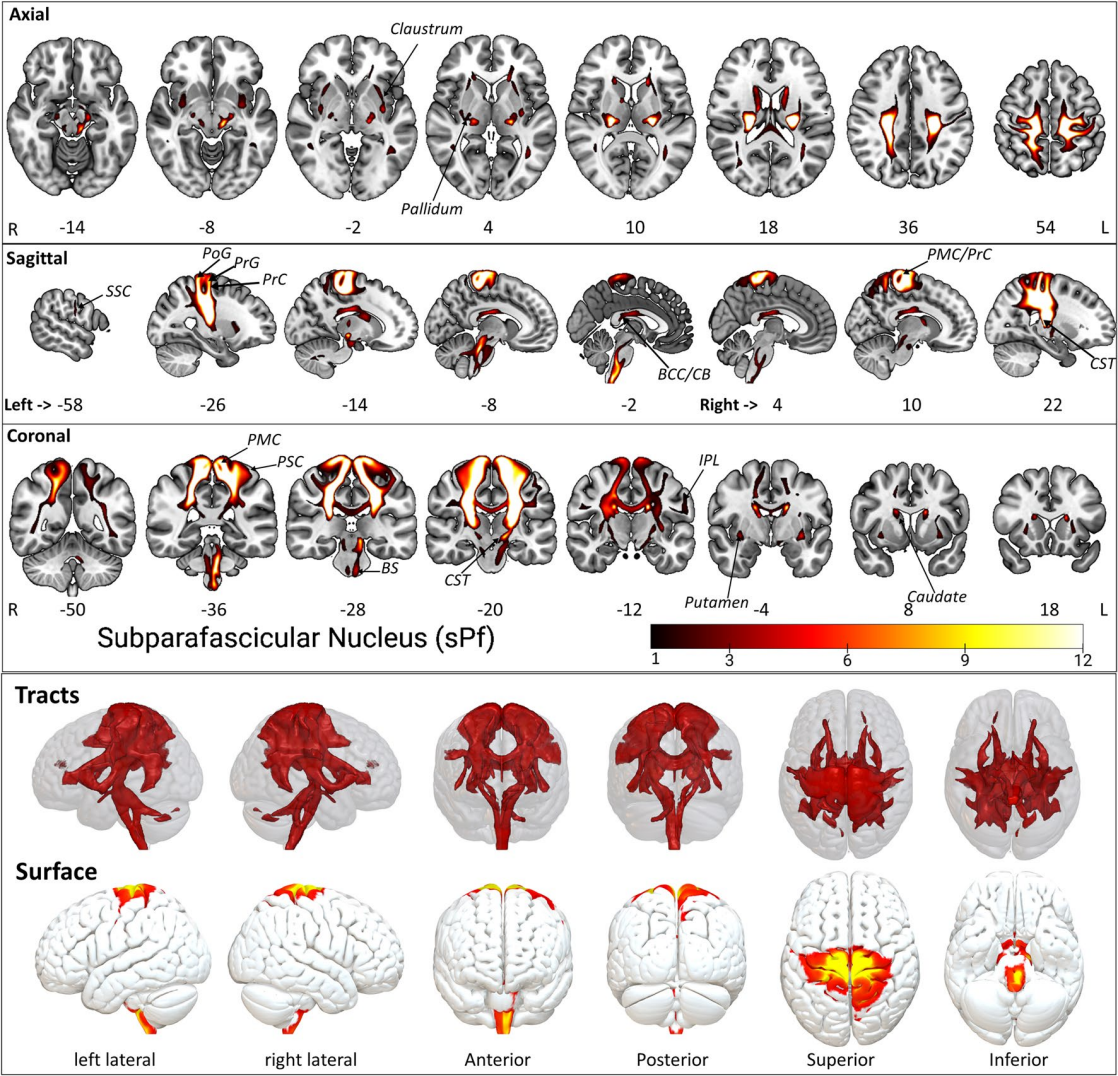
The sPf tract fixed effect maps in axial, coronal, sagittal, 3D rendered and surface views: *Connectivity:* The sPf white matter fiber shows a slight crossover with the STPs and PPTPs within the thalamus. The subcortical projections include caudate, putamen, and pallidum. sPF connections include the projections from the brainstem and CST. The cortical projections via CST and SLF enter the SSC, PSC, PMC, IC, PrG, PoG, superior parietal lobule 7PR, and inferior parietal lobule. Intrahemispheric pathway projected to the body of corpus callosum. *STPs* sensory thalamic projection site from oxford thalamus atlas, *PPTPs* posterior parietal thalamic projection site from oxford thalamus atlas, *CST* cortico spinal tract, *SLF* superior longitudinal fasciculus, *SSC* secondary somatosensory cortex, *PSC* primary somatosensory cortex, *PMC* primary motor cortex, *IC* insular cortex, *PrG* precentral gyrus, *PoG* postcentral gyrus. The detailed anatomical assignments are given in Supplementary Table 6.

**Figure 8:**
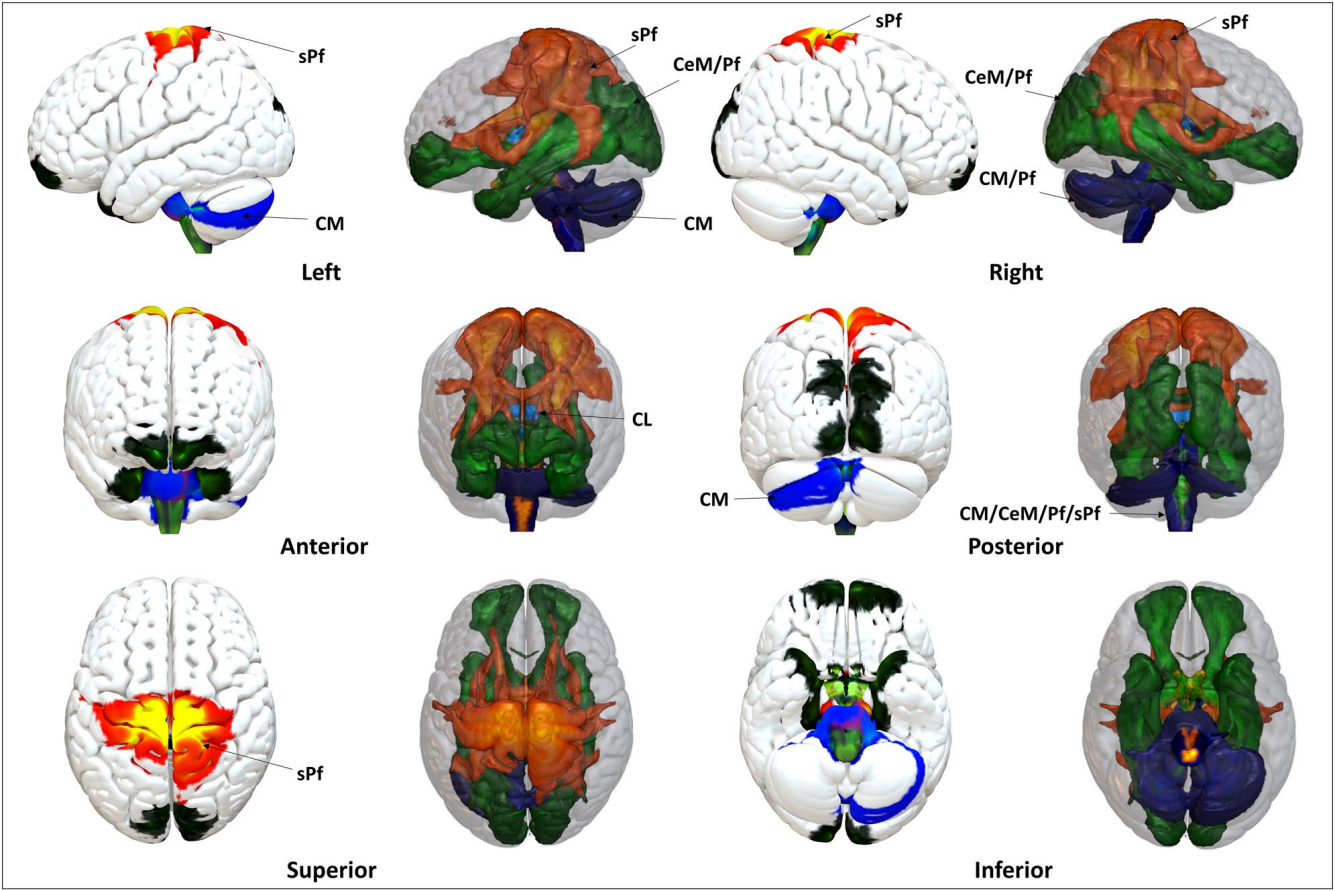
Intralaminar nuclei fixed effect map tract as 3D rendering and endpoints on the cortical surface: The ILN constitutes overlapping and specific projection sites in the brain. CeM and Pf displayed similar connected patterns to the visual, temporal, and frontal cortices. CM projected to the midbrain and cerebellum but not the superior cerebrum cortices. CL remains strictly confined to SC. sPf specifically comprises motor pathway projections from the brainstem, cerebellum, and motor cortex. Nuclei-specific connectivity anatomical assignments: *CL* The CL projections consist of SC and fornix. *CM:* The CM displayed connections to PMTPs, STPs, PTPs, MCP, SCP, cerebellum, BS, CST, Pd, and Ag. *CeM* The CeM projections consist of SCP, ICP, IOFF, aIOFFf, pIOFFf, MCP, CPCF, CSF, ILF, BS, HEC, UF, SPL-5M, CB, PTPs, SS, MGB, fornix, HS, Ag, OR, and the FC. *Pf* Pf projections are almost similar to the CeM projections but additionally show connectivity to the lateral occipital cortex, precuneus, and splenium of the corpus callosum. However, unlike the CeM projections, no intrathalamic connections to the PTPs were found. *sPf* sPf white matter tracts partly show connections with the STPs and PPTPs within the thalamus. The subcortical projections include the caudate, putamen, pallidum, and brainstem. The cortical projections project via CST and SLF to the SSC, PSC, PMC, IC, PrG, PoG, superior parietal lobule 7PR, and inferior parietal lobule, while an intra-hemispheric pathway projects to the corpus callosum. The detailed anatomical assignments are given in Supplementary Tables 2–11.

The resulting group fixed effect maps were visualized using the mricron package. The group fixed effect maps were minimally thresholded (thr 1) to remove weaker and spurious probabilities. The brain's axial, sagittal, and coronal views visualize each fixed-effect-map (Figs. 3, 4, 5, 6, 7, 8, Supplementary Figs. 1–6). The labeled 2D slices were shown in axial, sagittal, and coronal views. The connectivity on the cortical surface visualizes the endpoints of the touching tract volume mesh. The 3D tracts (rendering and surface) illustrations are arranged side to side in six different viewpoints, i.e., left, right, posterior, anterior, inferior, and superior. The rendering views were visualized using the surfice software package.

#### Anatomical atlas label search and assignments

The anatomical assignments of the fixed effect maps (thr 1) determined specific labels for cortical, subcortical, white-matter tracts, and cerebellar projections. The anatomical labeling procedure employed the Harvard–Oxford cortical-subcortical structural atlas^38–41^, the JHU white matter tractography atlas^42^, the Jülich histological atlas^43–45^, oxford thalamus atlas, subthalamic nucleus atlas, oxford manova striatal structural atlas, Human sensorimotor tracts label atlas, XTRACT HCP probabilistic tract atlas, and the cerebellar atlas in MNI152 space after normalization with FNIRT^46^ available within FSL^47^. The oxford thalamus atlas assigns the connectivity localization within the thalamus^48^**.** The detailed assignments of the brainstem nuclei relied on the brainstem navigator atlas^49^. In the results, the description of pathways and connections uses the words ('project/projected') to describe diffusion data-driven structural connectivity, which doesn’t distinguish between the incoming and outgoing connections to the individual ILN due to the underlying methodological limitations.

## Results

### Intralaminar nuclei of the thalamus

The ILN enveloping the medially located mediodorsal nucleus (MD) refers to assembling nuclear structures within the thalamus' internal medullary lamina (IML) (Fig. 2). The IML is a remarkably constructed myelinated fiber pathway in the center of the thalamus. It appears as a Y-shaped white stripe in axial sections and delimits the different thalamic territories that form the medial, lateral, and anterior groups of thalamic nuclei. In general, the ILN has been associated with the truncothalamic complex, as they constitute a major part of the so-called 'nonspecific' thalamocortical system that relays the activity of the brainstem reticular formation to widespread cerebral cortical areas. Depending on the referring anatomist^50–52^, the ILN can be divided into two or three groups (Fig. 2). The first is the central medial nucleus (CeM), located at the midline between the ILM and the mediodorsal nucleus (MD). The second is situated laterally in the anterior part of the IML and includes the paracentral and central lateral (CL) nuclei. The third expands posteriorly in splitting the IML and includes the posterior intralaminar, centre median (CM), and parafascicular (Pf) nuclei. Some authors^2,51^ further distinguish a small subparafascicular nucleus (sPf) within a splitting of the external medullary Iamina just ventral to the Pf proper.

### Structural connectivity

#### Central lateral nucleus CL

The CL is the largest intralaminar nucleus in the morel atlas^28^, revealing the most confined connectivity of all ILN***.*** The CL expands anteriorly to posterior, dorsally covering CeM, Pf, sPf, and CM (Fig. 2). The CL connectivity maps (Fig. 3, Supplementary Fig. 1, Supplementary Table 2) results show that the major CL pathway connects to the superior dorsal portion of the midbrain tectum, i.e., the superior colliculus (SC) and fornix. The SC consists of superficial visual layers and connects with the intralaminar nuclei^53,54^. In addition, CL possesses intrinsic thalamic connections to the prefrontal projection site (PTPs) and the temporal projection site in the Oxford thalamus atlas (TTPs). Interestingly, the inception of anterior thalamic radiation (ATR) also shows connectivity with CL. The Anterior and superior thalamic radiation show slight dominance in the left CL connectivity map in contrast to the right CL (Supplementary Table 10).

#### Centromedian/Centremedian nucleus CM

The CM is the second-largest nucleus in the ILN group. The CM is located in the central core, among other ILN, in sagittal view ventrally to CL, above Pf, sPf, and posterior to CeM (Fig. 2, Supplementary Fig. 1a,b). The CM projects to wider motor and sensory system brain areas, suggesting a key role in the motor system (Fig. 4, Supplementary Fig. 2, Supplementary Table 3). The CM shows connectivity via the medial and superior cerebellar peduncle (MCP, SCP) with the brainstem (BS) and cerebellar lobules, i.e., crus I, crus II, V, IX, I-IV, VIIb. The lobule I-IV and V are part of the somatotopic motor system^55^. However, connections to the other cerebellar lobules suggest a broader functional integration with working memory (crus-I/II) and multisensory integration (VIIb). Furthermore, CM connects to the corticospinal tract (CST); however, the CST does not reach the cortex. Interestingly, the CM connectivity to the amygdala superficial group (Ag) is found. We have noticed similar connections to the pallidum in line with these results.

The brain stem nuclei, i.e., raphe nucleus, periaqueductal gray, cuneiform nucleus, inferior colliculus, Inferior medullary reticular formation, inferior olivary nucleus, parabrachial nucleus, prabigeminal nucleus, mesencephalic reticular formation, pedunculopontine nucleus, sustantia nigra, vestibular nuclei, viscero-sensory-motor nuclei, and ventral tegmental area show prominent connectivity with CM (Supplementary Table 7). The cerebellar lobule Left Crus II shows a slightly higher overlap with Left CM, in contrast with Right Crus II (Supplementary Table 8).

#### Central medial nucleus CeM

The CeM is the third-largest nucleus within the ILN group. In the dorsal view, CeM is located below CL, next to the medial wall of the brain hemisphere (Fig. 2). In the sagittal view, the CeM locates itself at the anterior border and neighboring the posteriorly situated Pf (Fig. 2). The CeM projects (Fig. 5, Supplementary Fig. 3, Supplementary Table 4) to the anterior commissure (AC) and then further via the ATR to the orbito-frontal cortices, especially in the Brodmann areas (BA) 11. The tracts migrate from the AC to the medial temporal lobes, encircle the amygdala (Ag), and connect to the hippocampus gyrus. The CeM further projects subcortically to the pallidum, putamen, and caudate, as well as to the fornix and cingulum. Posteriorly, the tracts run along the optic radiations, merging with the inferior occipitofrontal fasciculus (IOFF). The posterior part of the IOFF projects to the calcarine fissure, exhibiting a thin connection to the medial and superior occipital lobes as well as to the cuneus and precuneus. A second adjacent connection of the posterior IOFF runs to the inferior longitudinal fasciculus (ILF), enabling connections to the fusiform gyrus, hippocampus, parahippocampal gyrus, Ag, and the middle inferior temporal lobe. Another coherent projection arises from the mid-anterior IOFF, connecting to the uncinate fasciculus (UF) and the temporal lobe. The caudal CeM projections include the inferior anterior fasciculus of the IOFF, inferior occipitofrontal fasciculus fragment (aIOFFf), posterior inferior occipitofrontal fasciculus fragment (pIOFFf), ILF, BS, hippocampus entorhinal cortex (HEC), uncinate fasciculus (UF), superior parietal lobule 5M (SPL-5M), callosal body (CB), sagittal stratum (SS: including ILF & IFOF), fornix, hippocampus subiculum (HS), Ag laterobasal group, optic radiation (OR) and the fusiform cortex (FC). Furthermore, CeM projects within the thalamus to the medial geniculate body (MGB) and PTPs. The inferior connections include the MCP, SCP, and inferior cerebellar peduncles (ICP). The cerebellar peduncles adjacent to the dentate nucleus show further fiber connections to specific medial cerebellar lobules, i.e., Crus II, I–IV, and via cortico pontocerebellum fibers (CPCF), corticospinal fibers (CSF), and pontine fibers to the spinal cord.

The brain stem nuclei, i.e., raphe nucleus, periaqueductal gray, Inferior medullary reticular formation, parabrachial nucleus, prabigeminal nucleus, mesencephalic reticular formation, pedunculopontine nucleus, vestibular nuclei, viscero-sensory-motor nuclei, and ventral tegmental area show prominent connectivity with CeM (Supplementary Table 7).

The Superior parietal lobule 7P shows slight dominance in the right CeM connectivity map compared to the left CeM (Supplementary Table 11).

#### Parafascicular nucleus Pf

The Pf, the second largest nucleus like CM, lies adjacent to the CeM at the posterior side (Fig. 2) and is sagittally located below the CL and neighboring CM. The Pf projections consist of SCP, ICP, IOFF, aIOFFf, pIOFFf, MCP, CPCF, CSF, ILF, BS, HEC, UF, SPL-5M, CB, PTPs, SS, MGB, fornix, HS, Ag, OR, and the FC (Fig. 6, Supplementary Fig. 4, Supplementary Table 5). The Pf projections are, moreover, similar to the CeM projections (Supplementary Text). The Pf shares most tracts of the CeM except exhibiting more extensive connections to the ILF, lateral occipital cortex, precuneus, and slightly to the splenium of the corpus callosum. Similar connections of the adjacent CeM and Pf suggest that they share identical thalamus peduncles/radiations and project to similar brain areas due to their spatial proximity.

Interestingly, both share connections to important brain areas, including visual, temporal, and frontal cortices. The latter are among other brain regions that contain significant nodes in the human default mode brain network. The CeM and Pf connectivity similarity possibly provides connectivity demands for the highly activated default mode network facilitating arousal, awareness, and other functions. The Visual cortex V1 BA17 shows slight dominance in the right Pf connectivity map compared to the left Pf (Supplementary Table 11).

The brain stem nuclei, i.e., raphe nucleus, parabrachial nucleus, prabigeminal nucleus, mesencephalic reticular formation, pedunculopontine nucleus, vestibular nuclei, and viscero-sensory-motor nuclei show prominent connectivity with Pf (Supplementary Table 7).

#### Subparafascicular nucleus sPf

The sPf is the smallest nucleus and lies as a tiny elliptical-shaped extended space under the Pf (Fig. 2). However, the sPf displays some unique connectivity patterns compared to other nuclei (Figs. 7, 8, Supplementary Figs. 5–7, Supplementary Table 6). The subcortical projections include caudate, putamen, and pallidum. These connections include the BS and corticospinal tract (CST). The cortical projections via the CST and superior longitudinal fasciculus (SLF) enter cortical areas, including the secondary somatosensory cortex (SSC), the primary somatosensory cortex (PSC), the primary motor cortex (PMC), insular cortex (IC), the precentral gyrus (PrG) and postcentral gyrus (PoG), the superior parietal 7PR and the inferior parietal lobule. Interestingly, most of these brain areas are also part of the broader somatosensory system, permitting motor and sensory computation as well as spatial orientation^56^ and awareness of the somatotopic events^57^. The superior parietal lobule cortical areas show slight dominance in the right sPf connectivity map compared to the left sPf (Supplementary Table 11).

The brain stem nuclei, i.e., Inferior olivary nucleus, and prabigeminal nucleus, pedunculopontine nucleus, show prominent connectivity with Pf (Supplementary Table 7).

#### ILN specific connectivity

The ILN connectivity maps showed partly overlapping but specific projection patterns (Fig. 8, Supplementary Figs. 6–7, Supplementary Tables 2–11). Overall, the connectivity map reveals that all ILN connect to the brainstem, where all sensory afferents enter the brain. The CeM and Pf displayed similar connectivity patterns to the brainstem, cerebellum, visual, and frontal cortices. The CM directly projects mainly to the brainstem and cerebellum. The CL remains strictly confined to connectivity with the SC in brainstem. The sPF specifically contains motor pathway projections from the brainstem, cerebellum to the motor cortices, possibly facilitating rapid motor planning, execution, and action.

## Discussion

The results gathered in this work reveal specific and partly overlapping connectivity patterns spanning a wide range of subcortical and cortical areas by utilizing high-resolution diffusion data in an HCP sample of 730 healthy subjects to determine the nuclei-specific connectivity of five ILN. The central medial nucleus (CeM) and the parafascicular nucleus (Pf) have particularly broad connectivity to the brainstem, cerebellum, subcortex, visual and frontal cortices, while the centromedian (CM) connects mainly to the subcortical motor system, including the brainstem and the cerebellum. The central lateral (CL) connects to the superior colliculus and fornix. The subparafascicular nucleus (sPF) presents specific projections to the basal ganglia, motor, somatosensory, parietal, and insular cortices. In short, the ILN offers overlapping and diverse connectivity patterns, suggesting variations in their functional involvement. The results of this research paint a picture of nuclei-specific ILN connections to subcortical and cortical areas, providing a deeper understanding of the intricacies of the thalamus.

In the discussion, the first section below compares the findings with the previous studies, followed by nuclei-specific connectivity in animal tracer studies. The tracer connectivity description elevates the understanding of the diffusion-tractography-driven ILN connectivity maps. The third section discusses brain-wide connectivity maps and their functional associations as the ILN has been implicated in various brain functions, i.e., conscious state, arousal, visual, sensorimotor, and attention^13,19,58^. The last section discusses the study's limitations and challenges.

### ILN connectivity

The ILN connectivity patterns demonstrate partly overlapping and nuclei-specific connections. The connectivity maps have shown that all ILN has prominent connections to the brainstem, highlighting the close relationship between the ILN and the brainstem, where all sensory information enters the brain. Remarkably, all ILN connects with the brainstem connections, which is important for numerous brain functions, including motor, sensory, arousal, and vigilance^16^. Despite being the largest ILN, CL shows a rather refined projection to the SC. While CM remains confined to the subcortical cerebellar and brainstem projections, the CeM and Pf connect to the frontal, visual, temporal, and subcortical brain regions, encompassing key areas of the default mode network nodes. The sPf outlines specific tracts to the somatosensory cortex encircling the sensorimotor network.

#### Comparison with previous diffusion and functional MRI studies

This study highlights the nuclei-specific detailed connectivity (Fig. 8, Supplementary Tables 2–11), in contrast to previous work by Jang and colleagues and Lambert and colleagues, who combined all intralaminar nuclei to perform a structural connectivity mapping of the ILN^22,23^. These studies^22,23^ fails to distinguish between the different nuclei of ILN. For instance, Jang et al. and colleagues used the Oxford thalamic atlas to delineate a single ILN mask containing all ILN. Using a single ILN mask that encircles all regions of ILN cannot be directly compared with our nuclei-specific connectivity maps. However, our nuclei-specific combined connectivity maps (Fig. 8, Supplementary Figs. 6, 7) reveal similarities as well as some distinct differences in contrast to combined ILN connectivity maps reported by Jang et al. and colleagues^22^. In particular, we found new connections to specific visual cortices, i.e., V1-V2, parts of the brainstem, and cerebellum lobules (I-IV, V, VIIb, Crus I, and Crus II). A significant difference also exists regarding the data quality of the HCP, the number of subjects, and the state-of-the-art analysis methodology, i.e., diffusion spectrum imaging/multishell reconstruction^59^.

Lambert and colleagues^23^ used euclidean distance to characterize probabilistic tractography distributions derived from diffusion MRI of 40 subjects from the HCP. Their study generated 12 feature maps to delineate individual thalamic nuclei, extracted tractography profiles for each and calculated the voxel-wise tractography gradients. Such feature maps do not delineate nuclei-specific maps of the intralaminar group. However, the combined midline-intralaminar feature map was found to have connections to the orbitofrontal cortex, entorhinal and calcarine cortices, as well as to the striatum, amygdala, and ventral mesencephalon^23^. Basile and colleagues^60^ performed in-vivo super-resolution track-density imaging using 210 subjects from the human connectome project. Study^60^ examined the structural and functional connectivity of combined masks of CM/Pf and MD/CL; therefore, it is not directly comparable to individual intralaminar connectivity patterns in our study^60^. However, combining the structural and functional connectivity of the CM/Pf complex to the middle and superior frontal gyri, supplementary motor, sensory regions, middle cingulate cortex, and insula aligns with our Pf connectivity (Fig. 6). Notably, the previous studies^22,23,60^ offer a basis for comparison and reliability of this study's observed connectivity patterns of the ILN group.

#### Alignment with animal studies

In animals, an anterograde tracer injection displays their terminal's detailed nuclei connectivity patterns and passing fibers communicating to other brain areas. Numerous tracer studies existed on mouse thalamic tracking^61^, macaque thalamic connectivity, and several animal anterogrades^62–68^. Using such a robust tracer technique, the CeM shows projections to the rat’s brainstem^69,70,70–72^. CeM also shows projections to the amygdala, putamen, caudate, and cerebellum^19^. In the cortical tracer studies^19^, the CeM projects cingulate cortices in rats, cats, and monkeys to the perirhinal cortex, the entorhinal cortex, the visual areas, and the claustrum. Also, the CeM shows widespread projections in the rats across the different cortical areas^73^. The CL projects to the rat's brainstem. Indirect projections via transthalamic fibers to the prefrontal and temporal cortices align with the reported animal work^19^. Remarkably, our results show CL projections to the SC in brainstem, as reported in ILN-SC studies^53,54^. The CM, Pf, and sPf's widespread connections arguably make sense due to their broader functional implications. Altogether, diffusion data-driven ILN connectivity maps broadly depict the connectivity described in animal studies. However, a detailed point-by-point comparison is unattainable, as the tracer data cannot be normalized in human space and only has limited access for comparison.

#### The brain-wide connectivity and functional associations

The centrally located ILN establishes interconnectivity within the thalamus, enabling highly privileged access to various cortical areas^2,74,75^. The integration and synchronization of multiple brain areas^76–78^ can result in a stream of consciousness^79^. All ILN projections align with the literature, suggesting their fundamental role in conscious processing and awareness. Thus, well-aligned with previous studies, the results depict ILN connectivity to the brainstem, basal ganglia, forebrain, and sensorimotor cortex^21,80–85^. The results revealed that the ILN cumulatively connects via other thalamic nuclei and subcortical pathways to a wide range of cortical areas, which align with the system-wide arousal circuitry^58^. The arousal circuitry encircles widespread connections, including the brainstem, thalamus, hypothalamus, basal forebrain, and cerebral cortex^58,86^. It is widely accepted that arousal is required to process visual attention^87^. The ILN resides next to the most prominent thalamic nuclei, i.e., the mediodorsal nucleus (MD), which primarily connects with the prefrontal cortices, and these activations result in the wakening of the animal.

Our results also found that the sPf nuclei project to motor, sensory, and parietal cortices. These findings agree with Jones's matrix-core theory of thalamic organization, in which the matrix nuclei (including ILN) serve as a binding locus with the cortex to achieve synchrony^13–15^ and integration to perforce motor, sensory, parietal, frontal, and visual projections.^88,89^. Dystonia of the intralaminar midline complex causes fixed eye deviation, thought disorder, postural and autonomic disturbances^90^. The CM-Pf nuclei's underlying functions are mainly related to arousal, attention, and sensorimotor functions^91^. Attention involves wider brain areas, i.e., cerebellar lobules and the temporal lobe, to facilitate attention. In our study, CeM and midline nuclei project to the cingulate; they seem to play a role in effectively processing tactile-input/nociceptive information^92,93^.

The ILN connections and their underline implicated functions in the literature are discussed in more detail below.

#### The brainstem is the most engaged projection site

All ILN reveal connectivity with the brainstem (Fig. 8, Supplementary Figs. 6, 7, Supplementary Table 7). This finding aligns well with the previous research work. The previous research work shows that the ILN receives extensive inputs from the brainstem^19,81,94–96^, as the ILN constitutes the dorsal pathway of the ascending reticular activating system of the brainstem to the cortex^83,97^. It is known from animal work that the brain alerts while performing an electric stimulation on the midbrain reticular formation and intralaminar nuclei. In humans, ILN shows activations during rest in an attention-demanding task implicating that the ILN and brainstem are important in arousal and vigilance^16^. The brainstem reticular formation covers most arousal-specific nuclei^58^, including *locus coeruleus,* raphe nuclei, and ascending arousal brainstem nodes^98,99^. The ILN receives inputs from most arousal-specific nuclei^58^, including *locus coeruleus,* raphe nuclei, and ascending arousal brainstem nodes^98,99^, and connects them with different cortical areas^100^.

#### Connectivity to sensorimotor cortices

Several studies show the CM and sPf connections with the basal ganglia, motor, and sensory cortices^18,19,22,69–72,74,75,101^. Similarly, we found CM and sPf projections in the subcortical and cortical sensorimotor networks. The electrical stimulation of ILN induces head motion, eventually increasing responses to visual stimuli^102–104^. In a similar line of evidence, the Parent and Hazrati^105^ work indicates that CM can effectively play an essential role in motor response modulation rather than sensory, visceral, emotional, or cognition-related functional processes. The motor modulation induces dopamine release from the striatum^106^, which seems reasonable for the CM-pallidum projections. Degeneration of caudal ILN nuclei results in progressive supranuclear palsy and Parkinson's disease^107^. The determined CM and sPf somatosensory connections align well with Henderson's study and play a part in motor control^94^ and associative-limbic motor functions^19^.

#### Connectivity to SC

According to Jones's matrix core theory, the CL nucleus is a matrix nucleus, and attention employs such nuclei for higher-order computation^13–15^. While the CL reveals connectivity with the SC. In coordination with the thalamic reticular nucleus, pulvinar nuclei, and other brain areas, the SC might play a significant role in orienting, attentional focusing, attention selection, and attention implementation^108–112^. The SC continuously constructs discrete visual retinotopic fields and connects them with the pulvinar and lateral geniculate nucleus. Our analysis found connections of the CL with the PTPs and TTPs, suggesting a structural path between the SC communication with the parietal and temporal lobe. This supports the idea that the superior colliculus needs input from multiple brain areas to enable continuous visual field mapping. The CL to SC-thalamic projections are part of the arousal system, a converged forebrain circuit that controls orienting, defense (fight or flight behavior), and sensory-motor integration^54^. Visual awareness requires ILN involvement^113,114^ as arousal directly correlates with pupil size, visual processing, on–off cortical dynamics^115,116^, and attention changes^87^. CL projects to the SC^54^ to visual areas^53^, motor and arousal areas^117^ to continuously shape the visual experience.

#### Connectivity to parietal cortices

Parietal cortices achieve sensorimotor integration by transforming visual maps into non-retinocentric coordinates through multisensory areas. For example, the multisensory parietal cortex transforms visual maps into non-retinocentric coordinates^118^. ILN connectivity with selected parietal cortices suggests their involvement with multisensory integration and the maintenance of multisensory integration and synchronization.

#### Connectivity to frontal cortices

The reticular formation connects to ILN, the basal forebrain, hypothalamus, and prefrontal cortices as areas are involved in arousal, control of attention, and sensorimotor function^19,21,83–85,85^. The previous work demonstrates that the partial infarctions of the ILN cause cognitive deficits^19^, resulting in decreased flexibility in the employment of cognitive strategies, i.e., dysexecutive syndrome^119^.

#### CeM connectivity facilitates arousal and sleep

The thalamus acts as a hub for sleep for subcortical and cortical inputs^11^ and contributes to slow sleep oscillations in humans^120^. The CeM and other brain areas also play a role in arousal studies^58^. Specific deep brain stimulation of midline/intralaminar nuclei interventions show heightened arousal, speech recovery, restored executive motor control, and improved feeding behavior after severe traumatic brain injury-induced minimally conscious state^21^. A recent study shows that the tonic and burst firing pattern of CeM neurons can modulate brain-wide cortical activity during sleep and provide dual control of sleep–wake states^11^. In the CeM, the connections with the frontal cortices, the brainstem, raphe nucleus, ventral striatum, VTA, and hypothalamus are neuronal substrates of sleep–wake states.

### Neuromodulation and intralaminar nuclei

The central lateral (CL) nuclei show reduced consciousness in macaques after deep brain stimulation (DBS)^121^. The centromedian (CM) is a target nucleus for generalized or multifocal seizures for the neuromodulatory treatment using deep brain stimulation^122–124^. The neuromodulation of CM-Pf complex using deep brain stimulation for Tourette’s syndrome is also an emerging target for treatment^125^. The DBS of Pf may modulate cognitive functions by inducing molecular-level gene expression changes in the prefrontal cortex^126^. The sPf stimulations show dopamine release modulation in the inferior colliculus of rats and are suggested to be involved in the auditory processing deficits associated with Parkinson's^127^. Stimulation of the CM in people with epilepsy leads to activation of diffuse, cortico-cortical evoked potentials^128^. The neuromodulation of the intralaminar nuclei using DBS may engage the nuclei-specific connected cortical and subcortical areas in various ways, leading to precise changes in behavior. For instance, the CM connects subcortically with almost all the basal ganglia nuclei, i.e., caudate, putamen, pallidum, substantia nigra, subthalamic nucleus, and cortically with sensorimotor, premotor^129,130^, and dorsolateral prefrontal cortex^131^. Therefore, a deeper understanding is warranted concerning the neuromodulation of each intralaminar nuclei and their combinations to further understand the impact on connected cortical brain regions. Such exploration may provide a more comprehensive understanding of the intralaminar nuclei's clinical implications, translational potential, and relevance in neurological and psychiatric conditions. It would also strengthen the validity of this study's observed structural connectivity of the intralaminar nuclei.

### Limitations

#### Cortico-thalamo-cortical feedforward and feedback communication

The complex cortico-thalamo-cortical feedforward and feedback interrelationships employ a layer-specific input and output mode^132^. However, due to methodological limitations, diffusion imaging cannot delineate such layer/column-specific details. The diffusion imaging captures coarser resolution and, therefore, cannot infer the layer/column-specific precise details like interlayer communicational architecture, axon collaterals, cortico-thalamic branching axons, extrathalamic axon branching to different cortical areas, and differentiation between the driver and modulator connections.

#### Erroneous estimations due to atlas-defined seed regions

Our results rely on histologically defined seed regions, which always include a bias. The parcellation of the thalamus is a challenging and unresolved question^133^. The atlas-based method depends on a limited number of post-mortem brains, therefore, cannot account for interindividual variabilities^134^. The atlas-defined regions can contain a mix of signals which may not be specific to the functional similarities. This mixing could become worse when multiple subjects are grouped after MNI normalization. Using a standard anatomical seed region does not account for internal nuclei architectonics that partially influences neighboring nuclei. These mixed signals are the main seed region for the diffusion and functional thalamo-cortical connectivity analysis and thus can lead to erroneous estimations^135,136^. In this study, the diffusion analysis uses the native subject space; only the fiber projections were transformed in the MNI space. The percentage volume of the left and right group fixed effect maps depicts a variable overlap (Supplementary Table 12). The CeM shows a high overlap with Pf, while CL shows only minimal overlap. We also noticed that the CM displays partly overlap with CeM, Pf, and sPf. Finally, a significantly higher overlap was observed for the Pf with CeM. In contrast, sPf shows a low overlap with other nuclei. All these different overlaps can be partly attributed to the bias of the atlas-defined seed regions, but cases of high probablity suggest reliable connectivity projections (Supplementary Figs. 13).

A single nucleus may have specific sub-regions, and they can display variance in their projections to achieve a precise finely-grained functional influence in the brain^64^. The nuclei sub-regions can only contain a few thousand neurons due to the smaller size of the nuclei. Therefore, their subregional projections and anatomical-specific localization are lacking due to resolution limits in MRI.

#### Structural connectivity and different tracking algorithms:

Different algorithms can be applied to infer structural connectivity from diffusion MRI data, such as probabilistic tracking, deterministic tracking, and unscented Kalman filter (UKF) tracking algorithms. These algorithms can potentially affect the experimental results when evaluating structural connectivity. In the study, we only used a probabilistic tracking algorithm considering the vast amount of data, which requires computational resources and extended disk space. However, it will be imperative and informative to validate the results using multiple methods to ensure the robustness of the findings in a future study. Additionally, it is important to consider the specific features of each algorithm when interpreting the results and to be aware of each method's potential limitations and sources of bias.

#### Structural connectivity and diffusion MRI of the brain

Due to a limited resolution of 1.25 mm isotropic, we capture the structural connectivity maps in a young, large healthy cohort. In addition, the MR coils usually yield a higher signal-to-noise ratio on the cortical ribbon than on the subcortical structures. However, as the spatial resolution and sequence optimization for subcortical structures improve, we may infer more specific fiber paths and their configurations^137^. For example, at the moment, we have no insights on the specific tangential connections up to the level of the gray matter, i.e., U-fibers. Furthermore, we do not speculate how the current diffusion tractography results correlate with the myloarchitecture of the brain. There also remains a poor understanding of whether the projections originate from the specific nuclei or come from other brain areas. We cannot observe such connectivity linkage due to limited resolution. Hopefully, future work in the field will provide high-resolution data and better methods to precisely delineate the described connectivity results.

The diffusion tractography can give an erroneous estimation of connections by having false positives or negatives^36,138^. The false positives can be partly corrected using methods like thresholding, visual inspection, comparison with previous literature, cross-validation, use of multiple algorithms and parameters, and statistical correction. The described connectivity maps use thresholding, visual inspection, and comparison with described connectivity patterns in literature. The statistical correction (FWE p < 0.05 and p < 0.001) (Supplementary Figs. 8–12, Supplement Table 13), probability maps (Supplementary Figs. 13), and streamline statistics (Supplementary Table 14) indicate that the ILN consists of a statistical reliable connectivity distribution. A recent study shows that 97% of possible connections exist in the mouse cortex^139^. This percentage may vary since humans have very different cortical architecture. Therefore, the described connectivity should be looked up with the methodological awareness of diffusion spectrum imaging and under a constrained measurement parameter setting, which can induce drastic differences in the results.

The study provides a comprehensive understanding of the structural connectivity of the intralaminar nuclei, but the reproducibility of these findings in clinical populations remains to be determined. More research is needed to confirm that these connections can be consistently observed in clinical data. However, we can increase the chances of reproducing these findings in clinical populations by using similar methods and data acquisition techniques to the HCP study.

In summary, the structural mapping in the brain anticipates better and more precise delineation of connectivity using high-resolution data, high-field MR advancement, better diffusion reconstruction, tractography, and empirically referenced in-vivo findings of the population-level histological work. Therefore, our findings and fiber distributions underlining the anatomical-wiring information remain tentative.

## Conclusion

The ILN structural connectivity suggests a critical nuclei group in the structural path from the brainstem and the cerebellum to specific cortical areas. They display overlapping and nuclei-specific connectivities to specific cortical-subcortical cerebellar and brainstem sites. The sPf connectivity appears as a key in the somatosensory processing unit, covering the brainstem, cerebellar areas, basal ganglia, and specific cortices. Interestingly, CM seems to be an essential component of the subcortical somatosensory system. The CM connections are similar to the sPf projections but remain confined to the subcortex. The CeM and Pf show similar connectivity projections with a slight variance. The CeM projections, compared to the Pf, show dense intrathalamic connectivities. The Pf displays slightly more spacious cortical connectivities in comparison with the CeM. However, both project to the visual, frontal, and temporal cortices and give access to some default mode network nodes. The CeM and Pf notably project through the subcortical system to ILF, UF, and IOFF, allowing broad access to brain areas and enabling visual and cognitive processing. It is worth noting that the CL shows a precise projection to the SC in the brainstem. The five ILN diffusion-defined connectivity maps span a wide range of subcortical and cortical areas. The findings align with the known structural core for various functional demands like arousal, emotion, cognition, sensory, vision, and motor processing. The described ILN connectivity relies on diffusion-driven analysis and does not directly describe the axonal path. However, knowing the anatomical connections in this study may facilitate the investigation of the potential role of ILN in healthy and disordered brains.

## Supplementary Information

Supplementary Information 1.

Supplementary Information 2.

## Data Availability

The datasets analyzed during the current study are available in the Human Connectome project repository (http://www.humanconnectomeproject.org/). The intralaminar structural connectivity maps depicted in the figures are available at 10.6084/m9.figshare.23713290.v1.

## Abbreviations

AC: Anterior commissure
aIOFFf: Anterior inferior occipito frontal fasciculus fragment
AG(g): Amygdala laterobasal + superficial group
ATR: Anterior thalamic radiation
BS: Brainstem
BCC: Body of corpus callosum
CeM: Central medial nucleus
CPCF: Cortico ponto cerebellar fibers
Crus I: Crus cerebelli I
Crus II: Crus cerebelli II
CSF: Cortico spinal fibers
CST: Cortico spinal tract
CB: Callosal body
FC: Fusiform cortex
Hp: Hippocampus
HS: Hippocampus subiculum
HEC: Hippocampus entorhinal cortex
ILN: Intralaminar nuclei
ICP: Inferior cerebellar peduncle
IC: Insular cortex
IOFF: Inferior occipito frontal fasciculus
ILF: Inferior longitudinal fasciculus
IPL: Inferior parietal lobule
MCP: Medial cerebellar peduncle
MGB: Medial geniculate body
OFC: Orbito-frontal cortex
OR: Optic radiation
pIOFFf: Posterior inferior occipito frontal fasciculus fragment (part)
PTPs: Prefrontal thalamic projection site (from oxford thalamic atlas)
PMTPs: Premotor thalamic projection site (from oxford thalamic atlas)
PPTPs: Posterior parietal thalamic projection site (from oxford thalamic atlas)
OTPs: Occipital thalamic projection site (from oxford thalamic atlas)
TTPs: Temporal thalamic projection site (from oxford thalamic atlas)
PRMTPs: Premotor thalamic projection site (from oxford thalamic atlas)
PD(Pd): Pallidum
PoG: Postcentral gyrus
PrG: Precentral gyrus
PrC: Premotor cortex
PMC: Primary motor cortex
PSC: Primary somatosensory cortex
SCP: Superior cerebellar peduncle
SPL-5M: Superior parietal lobule 5M
SS: Sagittal stratum (include ILF & IFOF)
SC: Superior colliculus (Superior dorsal portion of the tectum)
STPs: Sensory thalamic projection site (from oxford thalamic atlas)
SLF: Superior longitudinal fasciculus
SSC: Secondary somatosensory cortex
TTPs: Temporal thalamic projection site (from oxford thalamic atlas)
UF: Uncinate fasciculus
V1: Primary visual area
V2: Secondary visual area

## Code availability

The used diffusion toolbox (FSL FDT) is available within the FSL Software library. https://fsl.fmrib.ox.ac.uk/fsl/fslwiki. Tool to visualize the maps: BrainVoyager, Mricron https://www.nitrc.org/projects/mricron, fsleyes https://fsl.fmrib.ox.ac.uk/fsl/fslwiki/FSLeyes, and connectome workbench.

## References

[1] Jones, E. G. *The Thalamus 2 Volume Set*. (Cambridge University Press, 2007).

[2] Morel, A. *Stereotactic Atlas of the Human Thalamus and Basal Ganglia* (CRC Press, 2007).

[3] Nieuwenhuys, R., Voogd, J., Huijzen, C. V., Huijzen, C. van & Voogd, J. *The Human Central Nervous System* (Springer, 2008).

[4] Nowinski WL (1998). Anatomical targeting in functional neurosurgery by the simultaneous use of multiple Schaltenbrand-Wahren Brain atlas microseries. Stereotact. Funct. Neurosurg..

[5] Schaltenbrand, G., Wahren, W. & Hassler, R. *Atlas for Stereotaxy of the Human Brain* (Thieme, 1977).

[6] Albe-Fessard D, Besson JM (1973). Convergent thalamic and cortical projections: The non-specific system. Somatosensory System.

[7] Berkley KJ, Benoist JM, Gautron M, Guilbaud G (1995). Responses of neurons in the caudal intralaminar thalamic complex of the rat to stimulation of the uterus, vagina, cervix, colon, and skin. Brain Res..

[8] Matsumoto N, Minamimoto T, Graybiel AM, Kimura M (2001). Neurons in the thalamic CM-Pf complex supply striatal neurons with information about behaviorally significant sensory events. J. Neurophysiol..

[9] Peschanski M, Guilbaud G, Gautron M (1981). Posterior intralaminar region in rat: Neuronal responses to noxious and nonnoxious cutaneous stimuli. Exp. Neurol..

[10] Saalmann YB (2014). Intralaminar and medial thalamic influence on cortical synchrony, information transmission and cognition. Front. Syst. Neurosci..

[11] Gent TC, Bandarabadi M, Herrera CG, Adamantidis AR (2018). Thalamic dual control of sleep and wakefulness. Nat. Neurosci..

[12] Gent TC, Bassetti CL, Adamantidis AR (2018). Sleep-wake control and the thalamus. Curr. Opin. Neurobiol..

[13] Jones EG (2009). Synchrony in the interconnected circuitry of the thalamus and cerebral cortex. Ann. N. Y. Acad. Sci..

[14] Jones EG (2002). Thalamic circuitry and thalamocortical synchrony. Philos. Trans. R. Soc. Lond. B. Biol. Sci..

[15] Jones EG (2001). The thalamic matrix and thalamocortical synchrony. Trends Neurosci..

[16] Kinomura S, Larsson J, Gulyás B, Roland PE (1996). Activation by attention of the human reticular formation and thalamic intralaminar nuclei. Science.

[17] Liu X (2018). Subcortical evidence for a contribution of arousal to fMRI studies of brain activity. Nat. Commun..

[18] Metzger CD (2010). High field fMRI reveals thalamocortical integration of segregated cognitive and emotional processing in mediodorsal and intralaminar thalamic nuclei. Front. Neuroanat..

[19] Van der Werf YD, Witter MP, Groenewegen HJ (2002). The intralaminar and midline nuclei of the thalamus. Anatomical and functional evidence for participation in processes of arousal and awareness. Brain Res. Rev..

[20] Ward LM (2011). The thalamic dynamic core theory of conscious experience. Conscious. Cogn..

[21] Schiff ND (2007). Behavioural improvements with thalamic stimulation after severe traumatic brain injury. Nature.

[22] Jang SH, Lim HW, Yeo SS (2014). The neural connectivity of the intralaminar thalamic nuclei in the human brain: A diffusion tensor tractography study. Neurosci. Lett..

[23] Lambert C, Simon H, Colman J, Barrick TR (2017). Defining thalamic nuclei and topographic connectivity gradients in vivo. Neuroimage.

[24] Van Essen DC (2012). The Human Connectome Project: A data acquisition perspective. Neuroimage.

[25] Van Essen DC (2012). Cortical cartography and Caret software. Neuroimage.

[26] Sotiropoulos SN (2013). Advances in diffusion MRI acquisition and processing in the Human Connectome Project. Neuroimage.

[27] Glasser MF (2013). The minimal preprocessing pipelines for the Human Connectome Project. Neuroimage.

[28] Krauth A (2010). A mean three-dimensional atlas of the human thalamus: Generation from multiple histological data. Neuroimage.

[29] Jenkinson M, Bannister P, Brady M, Smith S (2002). Improved optimization for the robust and accurate linear registration and motion correction of brain images. Neuroimage.

[30] Jenkinson M, Smith S (2001). A global optimisation method for robust affine registration of brain images. Med. Image Anal..

[31] Leemans A, Jones DK (2009). The B-matrix must be rotated when correcting for subject motion in DTI data. Magn. Reson. Med..

[32] Andersson JLR, Skare S, Ashburner J (2003). How to correct susceptibility distortions in spin-echo echo-planar images: Application to diffusion tensor imaging. Neuroimage.

[33] Andersson JLR, Sotiropoulos SN (2016). An integrated approach to correction for off-resonance effects and subject movement in diffusion MR imaging. Neuroimage.

[34] Andersson JLR, Sotiropoulos SN (2015). Non-parametric representation and prediction of single- and multi-shell diffusion-weighted MRI data using Gaussian processes. Neuroimage.

[35] Jbabdi S, Sotiropoulos SN, Savio AM, Graña M, Behrens TEJ (2012). Model-based analysis of multishell diffusion MR data for tractography: How to get over fitting problems. Magn. Reson. Med..

[36] Behrens TEJ, Berg HJ, Jbabdi S, Rushworth MFS, Woolrich MW (2007). Probabilistic diffusion tractography with multiple fibre orientations: What can we gain?. Neuroimage.

[37] Hernández M (2013). Accelerating fibre orientation estimation from diffusion weighted magnetic resonance imaging using GPUs. PLoS ONE.

[38] Desikan RS (2006). An automated labeling system for subdividing the human cerebral cortex on MRI scans into gyral based regions of interest. Neuroimage.

[39] Frazier JA (2005). Structural brain magnetic resonance imaging of limbic and thalamic volumes in pediatric bipolar disorder. Am. J. Psychiatry.

[40] Goldstein JM (2007). Hypothalamic abnormalities in schizophrenia: Sex effects and genetic vulnerability. Biol. Psychiatry.

[41] Makris N (2006). Decreased volume of left and total anterior insular lobule in schizophrenia. Schizophr. Res..

[42] MRI Atlas of Human White Matter (2006). AJNR Am. J. Neuroradiol..

[43] Eickhoff SB (2007). Assignment of functional activations to probabilistic cytoarchitectonic areas revisited. Neuroimage.

[44] Eickhoff SB, Heim S, Zilles K, Amunts K (2006). Testing anatomically specified hypotheses in functional imaging using cytoarchitectonic maps. Neuroimage.

[45] Eickhoff SB (2005). A new SPM toolbox for combining probabilistic cytoarchitectonic maps and functional imaging data. Neuroimage.

[46] Diedrichsen J, Balsters JH, Flavell J, Cussans E, Ramnani N (2009). A probabilistic MR atlas of the human cerebellum. Neuroimage.

[47] Jenkinson M, Beckmann CF, Behrens TEJ, Woolrich MW, Smith SM (2012). FSL. NeuroImage.

[48] Johansen-Berg H (2005). Functional-anatomical validation and individual variation of diffusion tractography-based segmentation of the human thalamus. Cereb. Cortex.

[49] Bianciardi M (2015). Toward an in vivo neuroimaging template of human brainstem nuclei of the ascending arousal, autonomic, and motor systems. Brain Connect..

[50] Benarroch EE (2008). The midline and intralaminar thalamic nuclei: Anatomic and functional specificity and implications in neurologic disease. Neurology.

[51] Jones, E. G. *Thalamus 2 Vol. Set* (2007).

[52] Macchi G, Bentivoglio M, Jones EG, Peters A (1986). The thalamic intralaminar nuclei and the cerebral cortex. Sensory-Motor Areas and Aspects of Cortical Connectivity.

[53] Harting JK, Hall WC, Diamond IT, Martin GF (1973). Anterograde degeneration study of the superior colliculus in Tupaia glis: Evidence for a subdivision between superficial and deep layers. J. Comp. Neurol..

[54] Krout KE, Loewy AD, Westby GWM, Redgrave P (2001). Superior colliculus projections to midline and intralaminar thalamic nuclei of the rat. J. Comp. Neurol..

[55] Grodd W, Hülsmann E, Lotze M, Wildgruber D, Erb M (2001). Sensorimotor mapping of the human cerebellum: fMRI evidence of somatotopic organization. Hum. Brain Mapp..

[56] Karnath HO (1997). Spatial orientation and the representation of space with parietal lobe lesions. Philos. Trans. R. Soc. B.

[57] Evrard HC (2019). The organization of the primate insular cortex. Front. Neuroanat..

[58] Satpute AB, Kragel PA, Barrett LF, Wager TD, Bianciardi M (2018). Deconstructing arousal into wakeful, autonomic and affective varieties. Neurosci. Lett..

[59] Wedeen VJ (2008). Diffusion spectrum magnetic resonance imaging (DSI) tractography of crossing fibers. Neuroimage.

[60] Basile GA (2021). In vivo super-resolution track-density imaging for thalamic nuclei identification. Cereb. Cortex.

[61] Oh SW (2014). A mesoscale connectome of the mouse brain. Nature.

[62] Berendse HW, Groenewegen HJ (1991). Restricted cortical termination fields of the midline and intralaminar thalamic nuclei in the rat. Neuroscience.

[63] Berendse HW, Groenewegen HJ (1990). Organization of the thalamostriatal projections in the rat, with special emphasis on the ventral striatum. J. Comp. Neurol..

[64] Dolleman-Van Der Weel MJ, Witter MP (1996). Projections from the nucleus reuniens thalami to the entorhinal cortex, hippocampal field CA1, and the subiculum in the rat arise from different populations of neurons. J. Comp. Neurol..

[65] Groenewegen HJ, Berendse HW (1994). The specificity of the ‘nonspecific’ midline and intralaminar thalamic nuclei. Trends Neurosci..

[66] Wouterlood FG, Saldana E, Witter MP (1990). Projection from the nucleus reuniens thalami to the hippocampal region: Light and electron microscopic tracing study in the rat with the anterograde tracer Phaseolus vulgaris-leucoagglutinin. J. Comp. Neurol..

[67] Wright CI, Groenewegen HJ (1996). Patterns of overlap and segregation between insular cortical, intermediodorsal thalamic and basal amygdaloid afferents in the nucleus accumbens of the rat. Neuroscience.

[68] Wright CI, Groenewegen HJ (1995). Patterns of convergence and segregation in the medial nucleus accumbens of the rat: Relationships of prefrontal cortical, midline thalamic, and basal amygdaloid afferents. J. Comp. Neurol..

[69] Peschanski M, Besson J-M (1984). A spino-reticulo-thalamic pathway in the rat: An anatomical study with reference to pain transmission. Neuroscience.

[70] Vertes RP (1991). A PHA-L analysis of ascending projections of the dorsal raphe nucleus in the rat. J. Comp. Neurol..

[71] Vertes RP, Martin GF (1988). Autoradiographic analysis of ascending projections from the pontine and mesencephalic reticular formation and the median raphe nucleus in the rat. J. Comp. Neurol..

[72] Villanueva L, Desbois C, Le Bars D, Bernard JF (1998). Organization of diencephalic projections from the medullary subnucleus reticularis dorsalis and the adjacent cuneate nucleus: A retrograde and anterograde tracer study in the rat. J. Comp. Neurol..

[73] Herkenham M (1980). Laminar organization of thalamic projections to the rat neocortex. Science.

[74] Kaitz SS, Robertson RT (1981). Thalamic connections with limbic cortex. II. Corticothalamic projections. J. Comp. Neurol..

[75] Kaufman EFS, Rosenquist AC (1985). Afferent connections of the thalamic intralaminar nuclei in the cat. Brain Res..

[76] Fries P (2005). A mechanism for cognitive dynamics: Neuronal communication through neuronal coherence. Trends Cogn. Sci..

[77] Singer W (1999). Neuronal synchrony: A versatile code for the definition of relations?. Neuron.

[78] Ward LM (2003). Synchronous neural oscillations and cognitive processes. Trends Cogn. Sci..

[79] James W (1890). The Principles of Psychology.

[80] Haber SN, Calzavara R (2009). The cortico-basal ganglia integrative network: The role of the thalamus. Brain Res. Bull..

[81] Krout KE, Belzer RE, Loewy AD (2002). Brainstem projections to midline and intralaminar thalamic nuclei of the rat. J. Comp. Neurol..

[82] Laureys S (2000). Restoration of thalamocortical connectivity after recovery from persistent vegetative state. The Lancet.

[83] León-Domínguez U, Vela-Bueno A, Froufé-Torres M, León-Carrión J (2013). A chronometric functional sub-network in the thalamo-cortical system regulates the flow of neural information necessary for conscious cognitive processes. Neuropsychologia.

[84] Paus T (2000). Functional anatomy of arousal and attention systems in the human brain. Prog. Brain Res..

[85] Smythies J (1997). The functional neuroanatomy of awareness: With a focus on the role of various anatomical systems in the control of intermodal attention. Conscious. Cogn..

[86] Parvizi J, Damasio A (2001). Consciousness and the brainstem. Cognition.

[87] Engel TA (2016). Selective modulation of cortical state during spatial attention. Science.

[88] Mumford D (1991). On the computational architecture of the neocortex. Biol. Cybern..

[89] Mumford D (1992). On the computational architecture of the neocortex. II. The role of cortico-cortical loops. Biol. Cybern..

[90] Schiff, N. D., Frucht, S., Purpura, K. P. & Ruggiero, D. A. The crises of von economo A dystonia of the intralaminar-midline thalamic complex? *Society for Neuroscience Abstracts* https://eurekamag.com/research/035/865/035865019.php (1999).

[91] Eckert U (2011). Preferential networks of the mediodorsal nucleus and centromedian-parafascicular complex of the thalamus-A DTI tractography study. Hum. Brain Mapp..

[92] Rainville P, Duncan GH, Price DD, Carrier B, Bushnell MC (1997). Pain affect encoded in human anterior cingulate but not somatosensory cortex. Science.

[93] Vogt BA, Rosene DL, Pandya DN (1979). Thalamic and cortical afferents differentiate anterior from posterior cingulate cortex in the monkey. Science.

[94] Cornwall J, Phillipson OT (1988). Afferent projections to the dorsal thalamus of the rat as shown by retrograde lectin transport. II. The midline nuclei. Brain Res. Bull..

[95] Hallanger AE, Levey AI, Lee HJ, Rye DB, Wainer BH (1987). The origins of cholinergic and other subcortical afferents to the thalamus in the rat. J. Comp. Neurol..

[96] Newman DB, Ginsberg CY (1994). Brainstem reticular nuclei that project to the thalamus in rats: A retrograde tracer study. Brain. Behav. Evol..

[97] Daube, J. R., Reagan, T. J. & Sandok, B. A. *Medical Neurosciences: An Approach to Anatomy, Pathology, and Physiology by Systems and Levels*. (Little Brown & Co, 1986).

[98] Edlow BL (2012). Neuroanatomic connectivity of the human ascending arousal system critical to consciousness and its disorders. J. Neuropathol. Exp. Neurol..

[99] Edlow BL (2013). Disconnection of the ascending arousal system in traumatic coma. J. Neuropathol. Exp. Neurol..

[100] Steriade M, Glenn LL (1982). Neocortical and caudate projections of intralaminar thalamic neurons and their synaptic excitation from midbrain reticular core. J. Neurophysiol..

[101] Sakai ST, Tanaka D (1984). Contralateral corticothalamic projections from area 6 in the raccoon. Brain Res..

[102] Hunsperger RW, Roman D (1976). The integrative role of the intralaminar system of the thalamus in visual orientation and perception in the cat. Exp. Brain Res..

[103] Schlag J, Schlag-Rey M (1984). Visuomotor functions of central thalamus in monkey. II. Unit activity related to visual events, targeting, and fixation. J. Neurophysiol..

[104] Schlag-Rey M, Schlag J (1984). Visuomotor functions of central thalamus in monkey. I. Unit activity related to spontaneous eye movements. J. Neurophysiol..

[105] Parent A, Hazrati LN (1995). Functional anatomy of the basal ganglia. I. The cortico-basal ganglia-thalamo-cortical loop. Brain Res. Brain Res. Rev..

[106] Kilpatrick IC, Jones MW, Johnson BJ, Cornwall J, Phillipson OT (1986). Thalamic control of dopaminergic functions in the caudate-putamen of the rat: II. Studies using ibotenic acid injection of the parafascicular-intralaminar nuclei. Neuroscience.

[107] Henderson JM, Carpenter K, Cartwright H, Halliday GM (2000). Loss of thalamic intralaminar nuclei in progressive supranuclear palsy and Parkinson’s disease: Clinical and therapeutic implications. Brain.

[108] Grieve KL, Acuña C, Cudeiro J (2000). The primate pulvinar nuclei: Vision and action. Trends Neurosci..

[109] LaBerge, D. *Attentional Processing: The Brain’s Art of Mindfulness*. (Harvard University Press, 1995).

[110] Lovejoy LP, Krauzlis RJ (2010). Inactivation of primate superior colliculus impairs covert selection of signals for perceptual judgments. Nat. Neurosci..

[111] Shipp S (2004). The brain circuitry of attention. Trends Cogn. Sci..

[112] Zeman A (1996). Attentional processing. The brain’s art of mindfulness. J. Neurol. Neurosurg. Psychiatry.

[113] Maquet P (1996). Functional neuroanatomy of human rapid-eye-movement sleep and dreaming. Nature.

[114] Purpura K (1997). The thalamic intralaminar nuclei: A role in visual awareness. Neuroscientist.

[115] Poulet JFA (2014). Keeping an eye on cortical states. Neuron.

[116] Reimer J (2014). Pupil fluctuations track fast switching of cortical states during quiet wakefulness. Neuron.

[117] Fisher SD, Reynolds JNJ (2014). The intralaminar thalamus: An expressway linking visual stimuli to circuits determining agency and action selection. Front. Behav. Neurosci..

[118] Sereno MI, Huang R-S (2014). Multisensory maps in parietal cortex. Curr. Opin. Neurobiol..

[119] Van der Werf YD, Witter MP, Uylings HB, Jolles J (2000). Neuropsychology of infarctions in the thalamus: A review. Neuropsychologia.

[120] Gemignani A (2012). Thalamic contribution to Sleep Slow Oscillation features in humans: A single case cross sectional EEG study in Fatal Familial Insomnia. Sleep Med..

[121] Redinbaugh MJ (2022). Thalamic deep brain stimulation paradigm to reduce consciousness: Cortico-striatal dynamics implicated in mechanisms of consciousness. PLOS Comput. Biol..

[122] Cukiert A, Cukiert CM, Burattini JA, Mariani PP (2020). Seizure outcome during bilateral, continuous, thalamic centromedian nuclei deep brain stimulation in patients with generalized epilepsy: A prospective, open-label study. Seizure Eur. J. Epilepsy.

[123] Vetkas A (2022). Deep brain stimulation targets in epilepsy: Systematic review and meta-analysis of anterior and centromedian thalamic nuclei and hippocampus. Epilepsia.

[124] Fisher RS (2023). Deep brain stimulation of thalamus for epilepsy. Neurobiol. Dis..

[125] Testini P, Min H-K, Bashir A, Lee KH (2016). Deep brain stimulation for Tourette’s syndrome: The case for targeting the thalamic centromedian-parafascicular complex. Front. Neurol..

[126] Fernández-Cabrera MR (2017). Parafascicular thalamic nucleus deep brain stimulation decreases NMDA receptor GluN1 subunit gene expression in the prefrontal cortex. Neuroscience.

[127] Batton AD, Blaha CD, Bieber A, Lee KH, Boschen SL (2018). Stimulation of the subparafascicular thalamic nucleus modulates dopamine release in the inferior colliculus of rats. Synapse.

[128] Zumsteg D, Lozano AM, Wieser HG, Wennberg RA (2006). Cortical activation with deep brain stimulation of the anterior thalamus for epilepsy. Clin. Neurophysiol..

[129] Ilyas A, Pizarro D, Romeo AK, Riley KO, Pati S (2019). The centromedian nucleus: Anatomy, physiology, and clinical implications. J. Clin. Neurosci..

[130] Sadikot AF, Rymar VV (2009). The primate centromedian–parafascicular complex: Anatomical organization with a note on neuromodulation. Brain Res. Bull..

[131] Le Reste P-J, Haegelen C, Gibaud B, Moreau T, Morandi X (2016). Connections of the dorsolateral prefrontal cortex with the thalamus: A probabilistic tractography study. Surg. Radiol. Anat..

[132] Usrey WM, Sherman SM (2018). Corticofugal circuits: Communication Lines from the cortex to the rest of the brain. J. Comp. Neurol..

[133] García-Cabezas MÁ, Pérez-Santos I, Cavada C (2023). Mapping the primate thalamus: Historical perspective and modern approaches for defining nuclei. Brain Struct. Funct..

[134] Webster, K. E. *Variations and Connections of the Human Thalamus, Part 1 (The Nuclei and Cerebral Connections of the Human Thalamus) and Part 2 (Variations of the Human Diencephalon): By JM Van Buren and RC Borke, xxii+ 587 pages, 98 illustrations, 179 Plates, 3 Tables, Springer-Verlag, Berlin, Heidelberg, New York, 1972, in 2 Parts, Not Sold Separately, DM 670.00; US $273.40* (Elsevier, 1974).

[135] Gordon EM (2016). Generation and evaluation of a cortical area parcellation from resting-state correlations. Cereb. Cortex.

[136] Smith SM (2011). Network modelling methods for FMRI. Neuroimage.

[137] Turner R, De Haan D, Mahfoud T, McLean S, Rose N (2017). Bridging the gap between system and cell: The role of ultra-high field MRI in human neuroscience. Progress in Brain Research.

[138] Parker GJM, Alexander DC (2005). Probabilistic anatomical connectivity derived from the microscopic persistent angular structure of cerebral tissue. Philos. Trans. R. Soc. Lond. B Biol. Sci..

[139] Gămănuţ R (2018). The mouse cortical connectome, characterized by an ultra-dense cortical graph, maintains specificity by distinct connectivity profiles. Neuron.

