# Supplementary material for: The structural connectivity mapping of the intralaminar thalamic nuclei
Source: Sci Rep. 2023 Jul 24;13:11938. doi: 10.1038/s41598-023-38967-0 (PMC10366221; doi:10.1038/s41598-023-38967-0)
Supplement: Supplementary file 1 — Supplementary Information 1. [file 41598_2023_38967_MOESM1_ESM.pdf]

SUPPLEMENTARY INFO

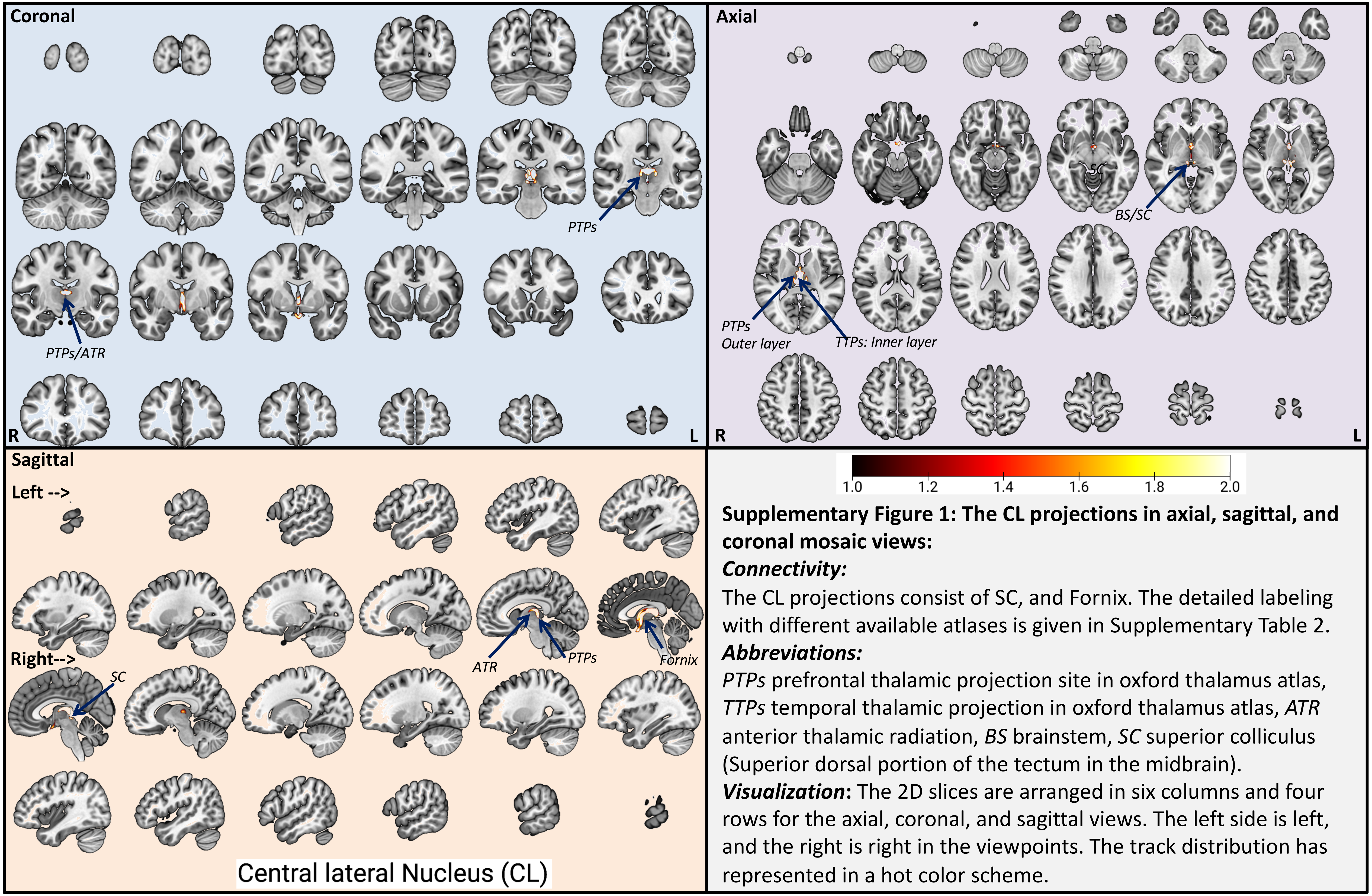

Supplementary Figure 1: The CL projections in axial, sagittal, and coronal mosaic views:

Connectivity:

The CL projections consist of SC, and Fornix. The detailed labeling with different available atlases is given in Supplementary Table 2.

Abbreviations:

PTPs prefrontal thalamic projection site in oxford thalamus atlas, *TTPs* temporal thalamic projection in oxford thalamus atlas, *ATR* anterior thalamic radiation, *BS* brainstem, *SC* superior colliculus (Superior dorsal portion of the tectum in the midbrain).

***Visualization*:** The 2D slices are arranged in six columns and four rows for the axial, coronal, and sagittal views. The left side is left, and the right is right in the viewpoints. The track distribution has represented in a hot color scheme.

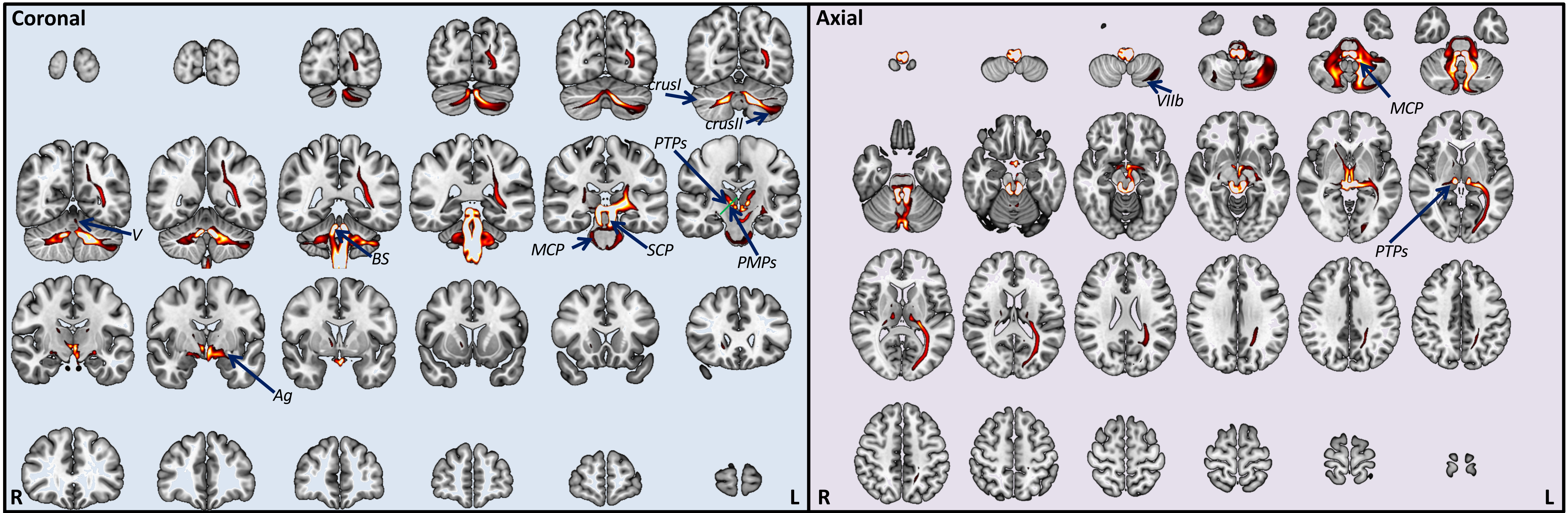

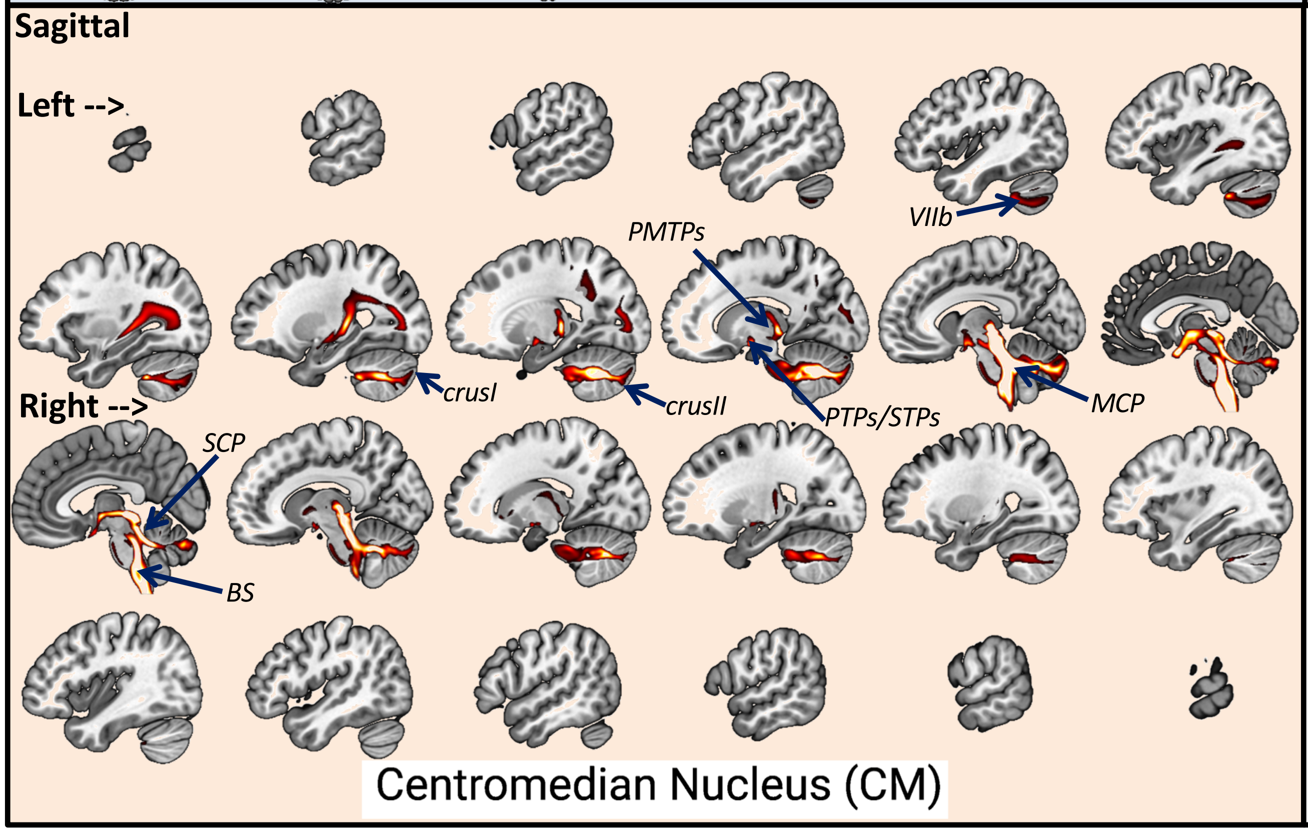

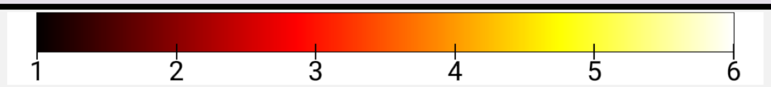

Supplementary Figure 2: The CM projections in axial, sagittal, and coronal mosaic views:

Connectivity:

The detailed labeling with different available atlases is given in Supplementary Table 3. The CM displayed connections to PMTPs, STPs, PTPs, MCP, SCP, cerebellar lobules (I-IV, V, VIIb, IX, CrusI, CrusII), BS, CST, Pd, and Ag.

Abbreviations:

PMTPs premotor thalamic projection site in oxford thalamus atlas, *STPs* sensory thalamic projection site in oxford thalamus atlas, *PTPs* prefrontal thalamic projection site in oxford thalamus atlas, *MCP* medial cerebellar peduncle, *SCP* superior cerebellar peduncle, *BS* brainstem, *CST* pre-corticospinal tract site, *Pd* pallidum, *Ag* amygdala superficial group.

***Visualization*:** The 2D slices are arranged in six columns and four rows for the axial, coronal, and sagittal views. The left side is left, and the right is right in the viewpoints. The track distribution has represented in a hot color scheme.

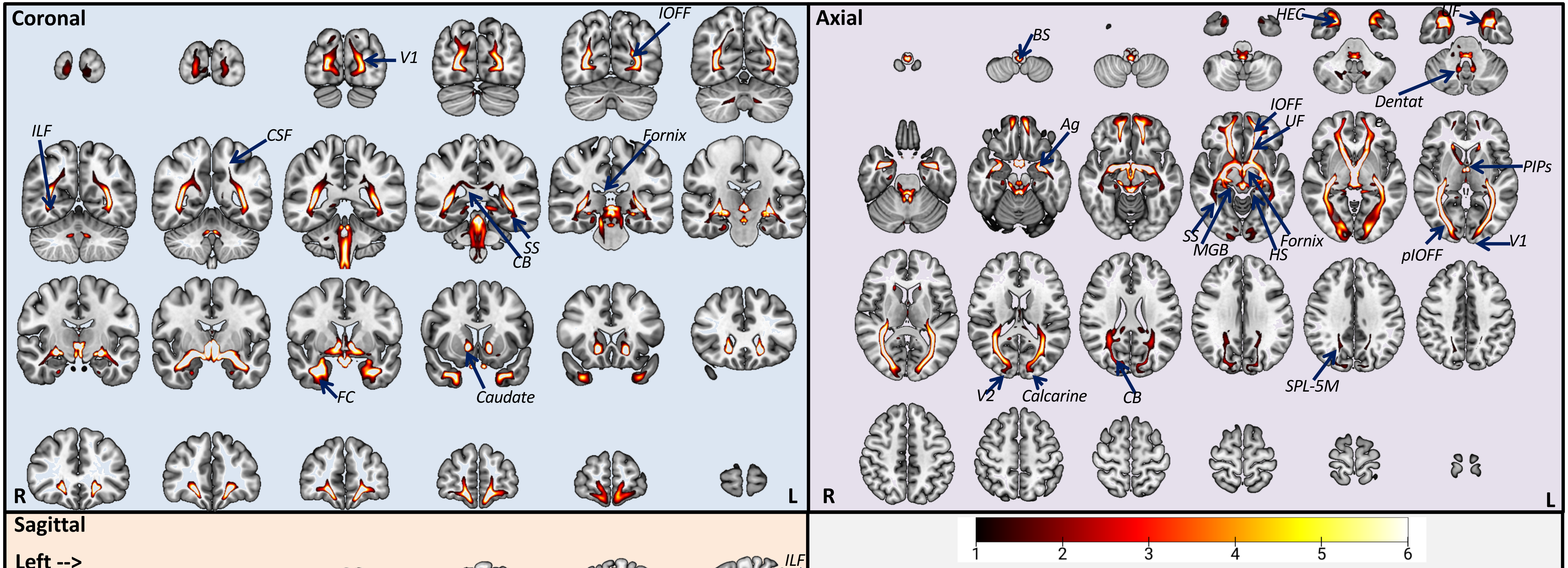

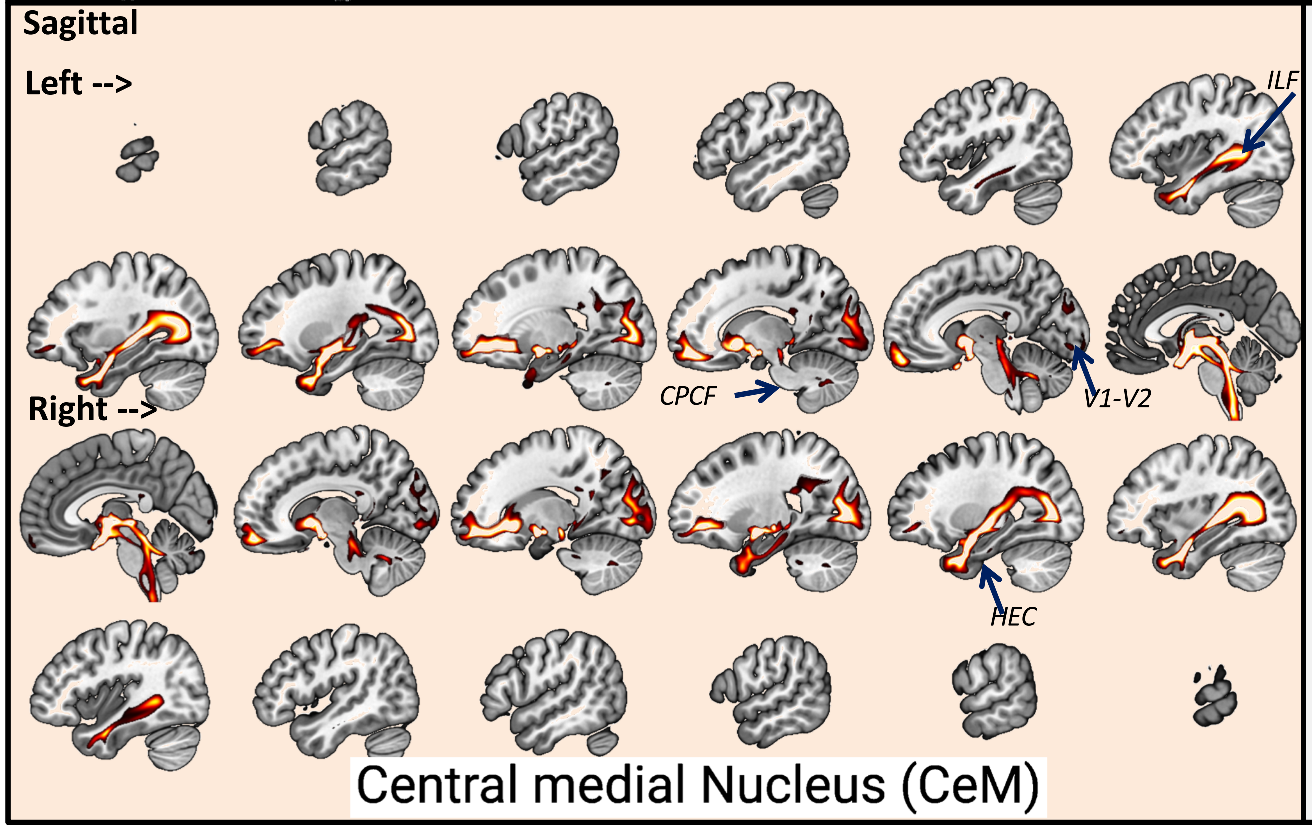

Supplementary Figure 3: The CeM projections in axial, sagittal, and coronal mosaic views:

Connectivity*:* The detailed labeling with different available atlases is given in Supplementary Table 4. The CeM projections consist of SCP, ICP, IOFF, aIOFFf, pIOFFf, MCP, CPCF, CSF, ILF, BS, HEC, UF, SPL-5M, CB, PTPs, SS, MGB, fornix, HS, Ag, OR, and the FC.

Abbreviations: *SCP* superior cerebellar peduncle, *ICP* inferior cerebellar peduncle, *IOFF* inferior occipito frontal fasciculus, *aIOFFf* anterior inferior occipito frontal fasciculus fragment, *pIOFFf* posterior inferior occipito frontal fasciculus fragment, *MCP* medial cerebellar peduncle, *CPCF* cortico ponto cerebellar fibers, *CSF* cortico spinal fibers, *ILF* inferior longitudinal fasciculus, *HEC* hippocampus entorhinal cortex, *BS* brainstem, *UF* uncinate fasciculus, *SPL-5M* superior parietal lobule 5M, *CB* callosal body, *SS* sagittal stratum (includes ILF & IFOF), *MGB* medial geniculate body, *HS* hippocampus subiculum, *Ag* amygdala laterobasal group, OR optic radiation, *FC* fusiform cortex.

Visualization: The 2D slices are arranged in six columns and four rows for the axial, coronal, and sagittal views. The left side is left, and the right is right in the viewpoints. The track distribution has represented in a hot color scheme.

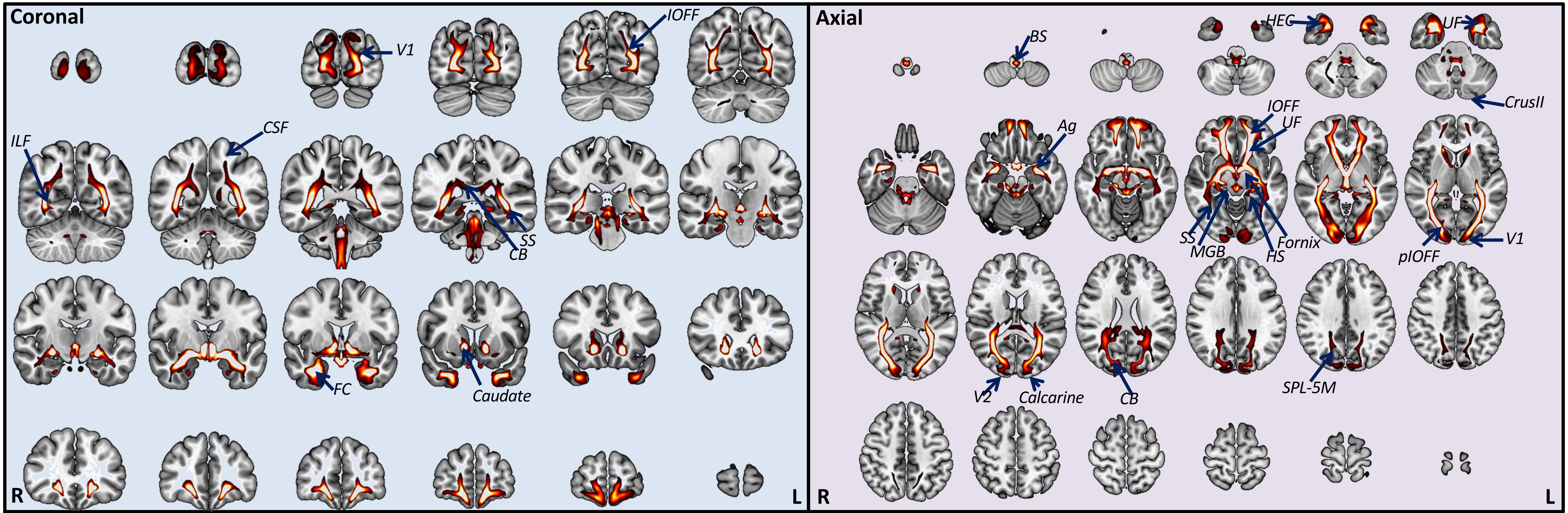

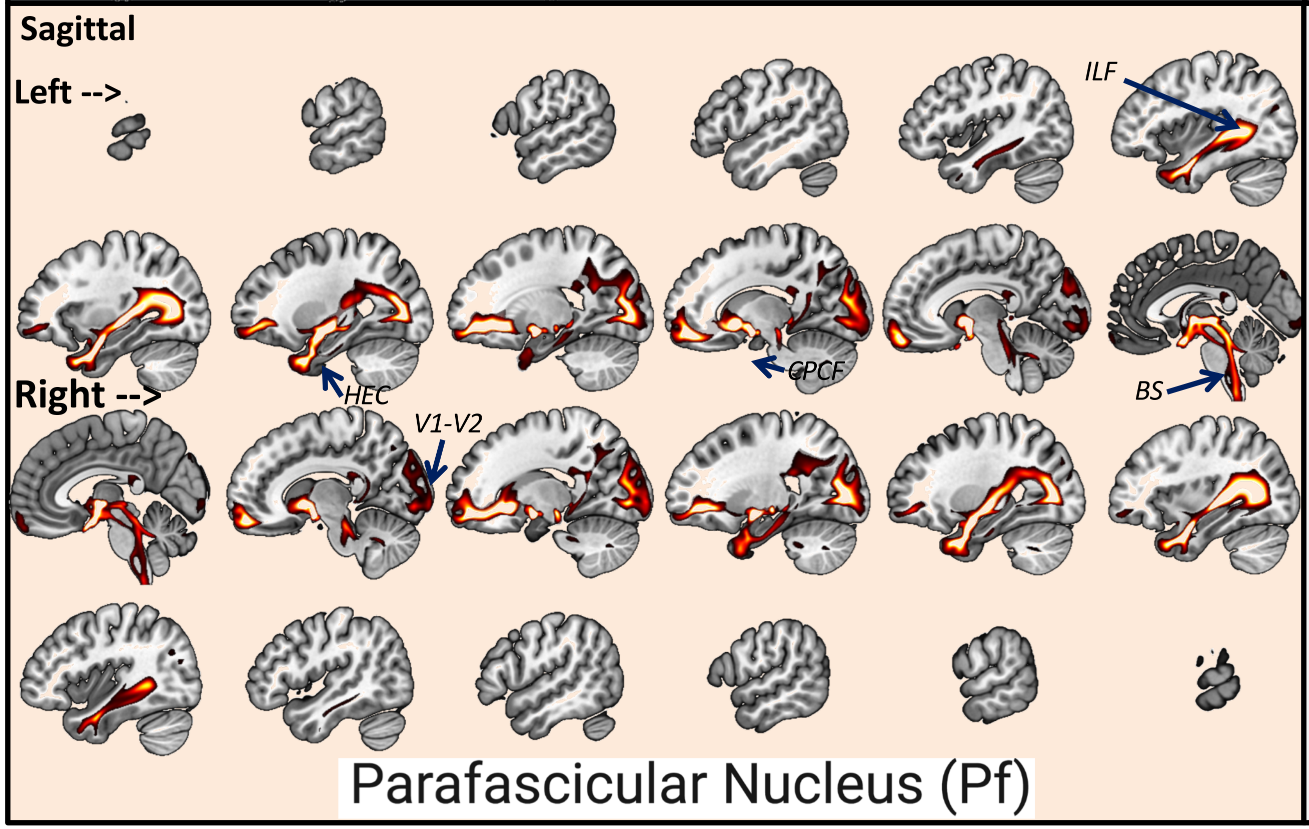

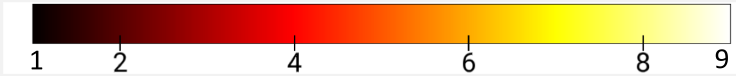

Supplementary Figure 4: The Pf projections in axial, sagittal, and coronal mosaic views: Connectivity:: The detailed labeling with different available atlases is given in Supplementary Table 5. The Pf projections are similar to the CeM projections except for a slightly dominated connectivity distribution into the Lateral occipital cortex, precuneus, and splenium of the corpus callosum.

Abbreviations: SCP superior cerebellar peduncle, ICP inferior cerebellar peduncle, IOFF inferior occipito frontal fasciculus, aIOFFf anterior inferior occipito frontal fasciculus fragment, pIOFFf posterior inferior occipito frontal fasciculus fragment, MCP medial cerebellar peduncle, CPCF cortico pontocerebellar fibers, CSF cortico spinal fibers, ILF inferior longitudinal fasciculus, HEC hippocampus entorhinal cortex, BS brainstem, UF uncinate fasciculus, SPL-5M superior parietal lobule 5M, CB callosal body, SS sagittal stratum (includes ILF & IFOF), MGB medial geniculate body, HS hippocampus subiculum, Ag amygdala laterobasal group, OR optic radiation, FC fusiform cortex, SS sagittal stratum (include ILF & IFOF) .

Visualization: The 2D slices are arranged in six columns and four rows for the axial, coronal, and sagittal views. The left side is left, and the right is right in the viewpoints. The track distribution has represented in a hot color scheme.

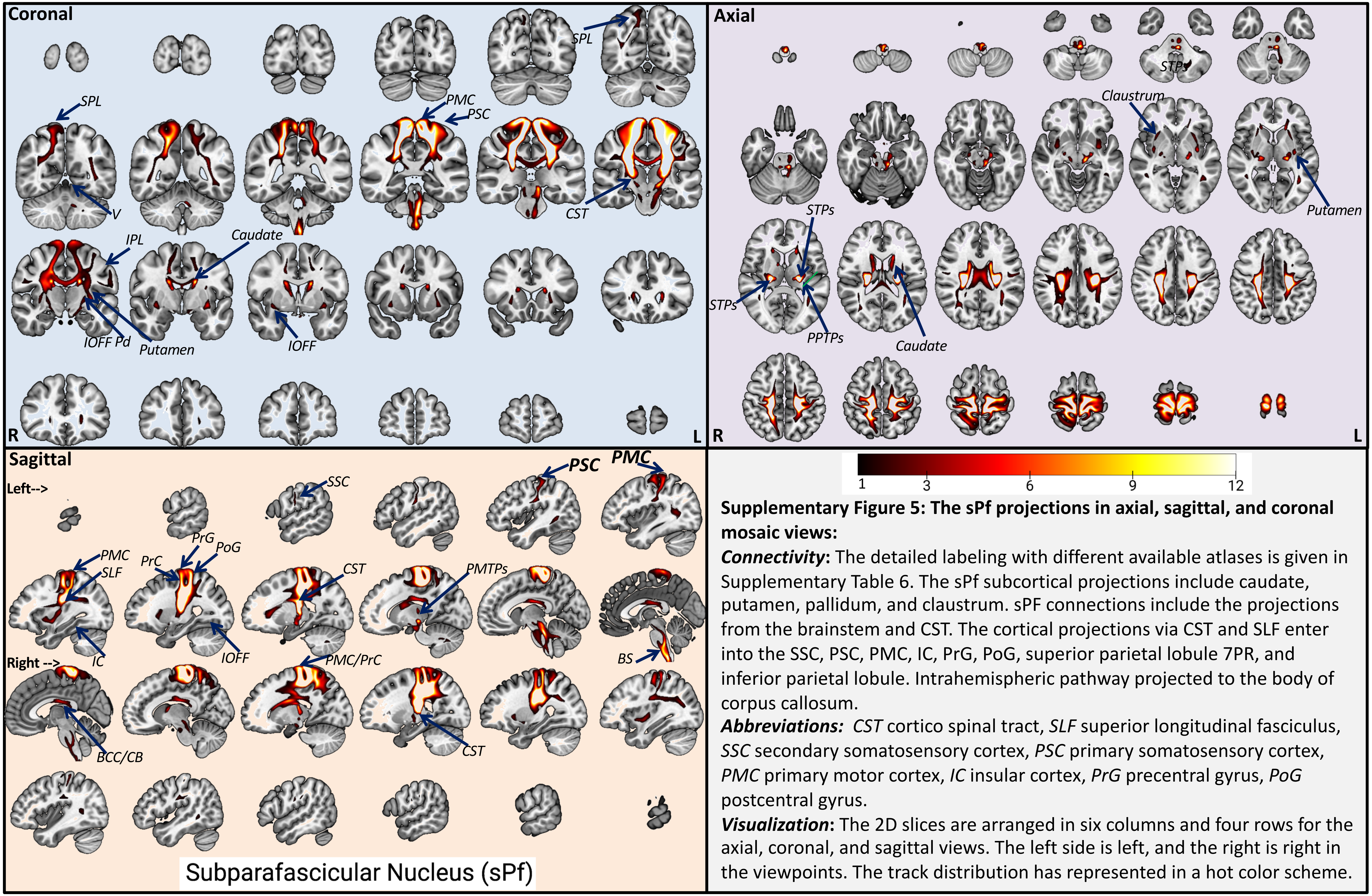

Supplementary Figure 5: The sPf projections in axial, sagittal, and coronal mosaic views:

Connectivity: The detailed labeling with different available atlases is given in Supplementary Table 6. The sPf subcortical projections include caudate, putamen, pallidum, and claustrum. sPF connections include the projections from the brainstem and CST. The cortical projections via CST and SLF enter into the SSC, PSC, PMC, IC, PrG, PoG, superior parietal lobule 7PR, and inferior parietal lobule. Intrahemispheric pathway projected to the body of corpus callosum.

Abbreviations: *CST* cortico spinal tract, *SLF* superior longitudinal fasciculus, *SSC* secondary somatosensory cortex, *PSC* primary somatosensory cortex, *PMC* primary motor cortex, *IC* insular cortex, *PrG* precentral gyrus, *PoG* postcentral gyrus.

Visualization: The 2D slices are arranged in six columns and four rows for the axial, coronal, and sagittal views. The left side is left, and the right is right in the viewpoints. The track distribution has represented in a hot color scheme.

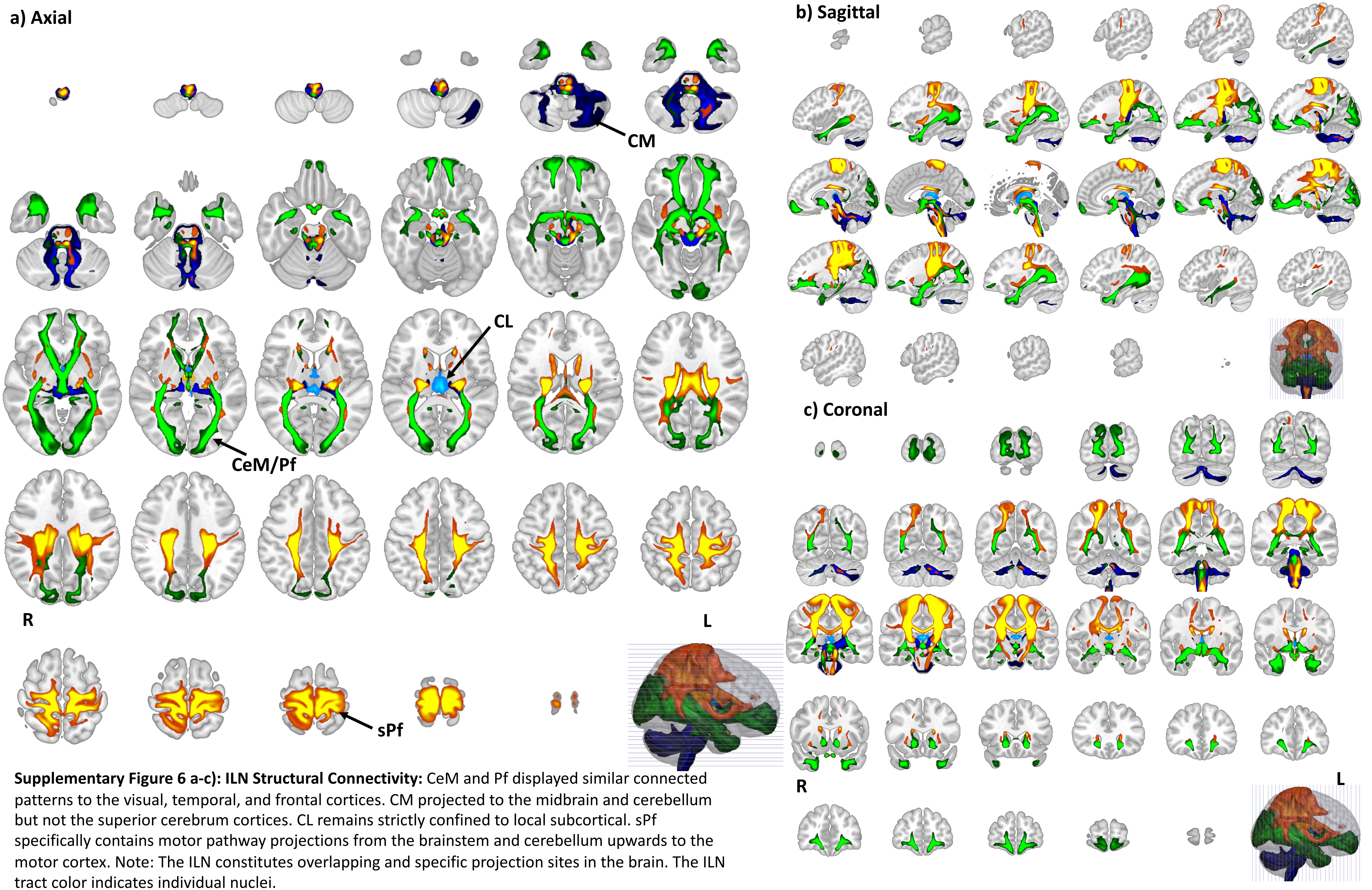

Supplementary Figure 6 a-c): ILN Structural Connectivity: CeM and Pf displayed similar connected patterns to the visual, temporal, and frontal cortices. CM projected to the midbrain and cerebellum but not the superior cerebrum cortices. CL remains strictly confined to local subcortical. sPf specifically contains motor pathway projections from the brainstem and cerebellum upwards to the motor cortex. Note: The ILN constitutes overlapping and specific projection sites in the brain. The ILN tract color indicates individual nuclei.

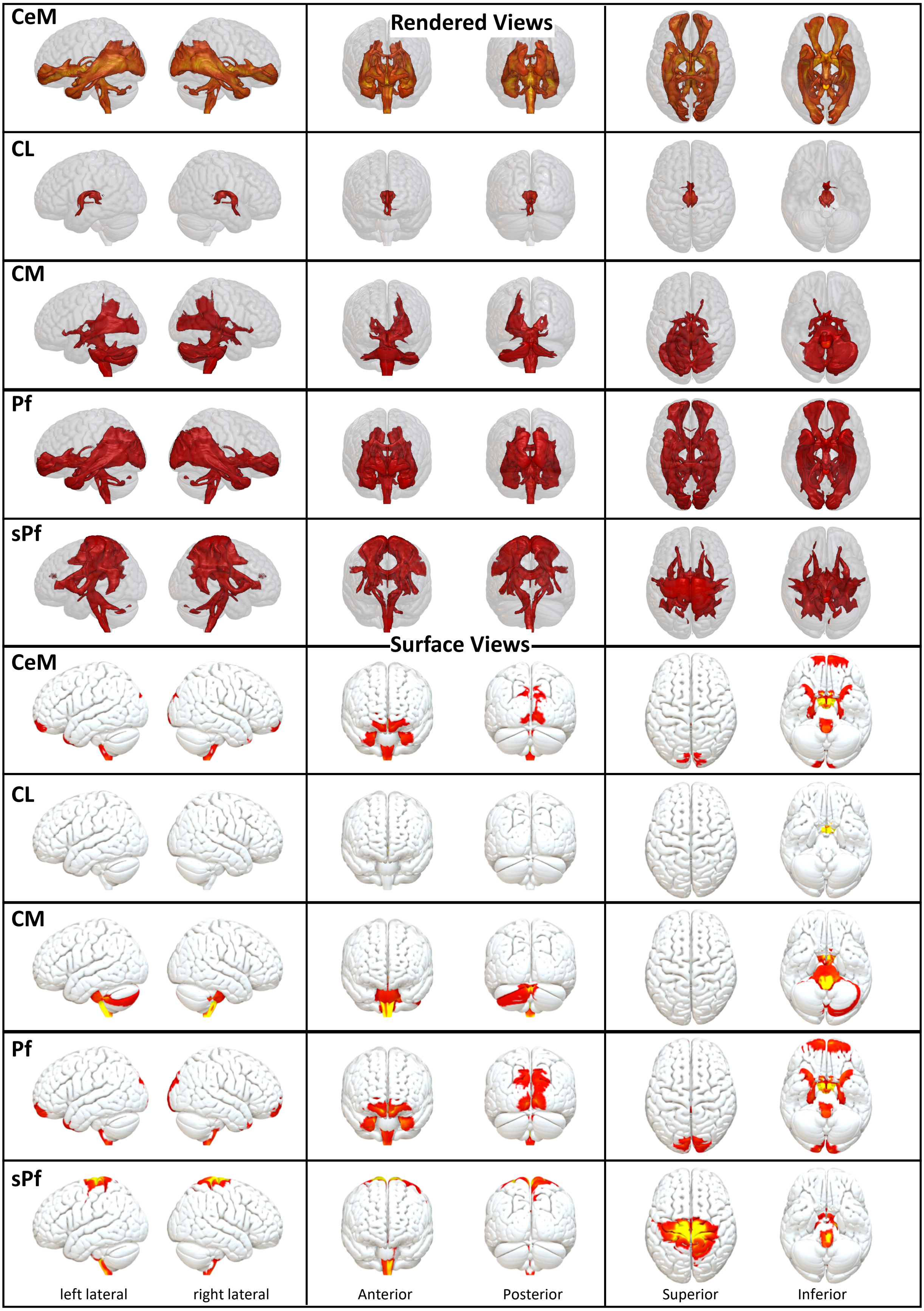

Supplementary Figure 7: ILN connectivity – 3D rendering and surface viewpoints, i.e., left, right, posterior, anterior, inferior, superior. The detailed connectivity of each nuclei is in Supplement Table 2-11.

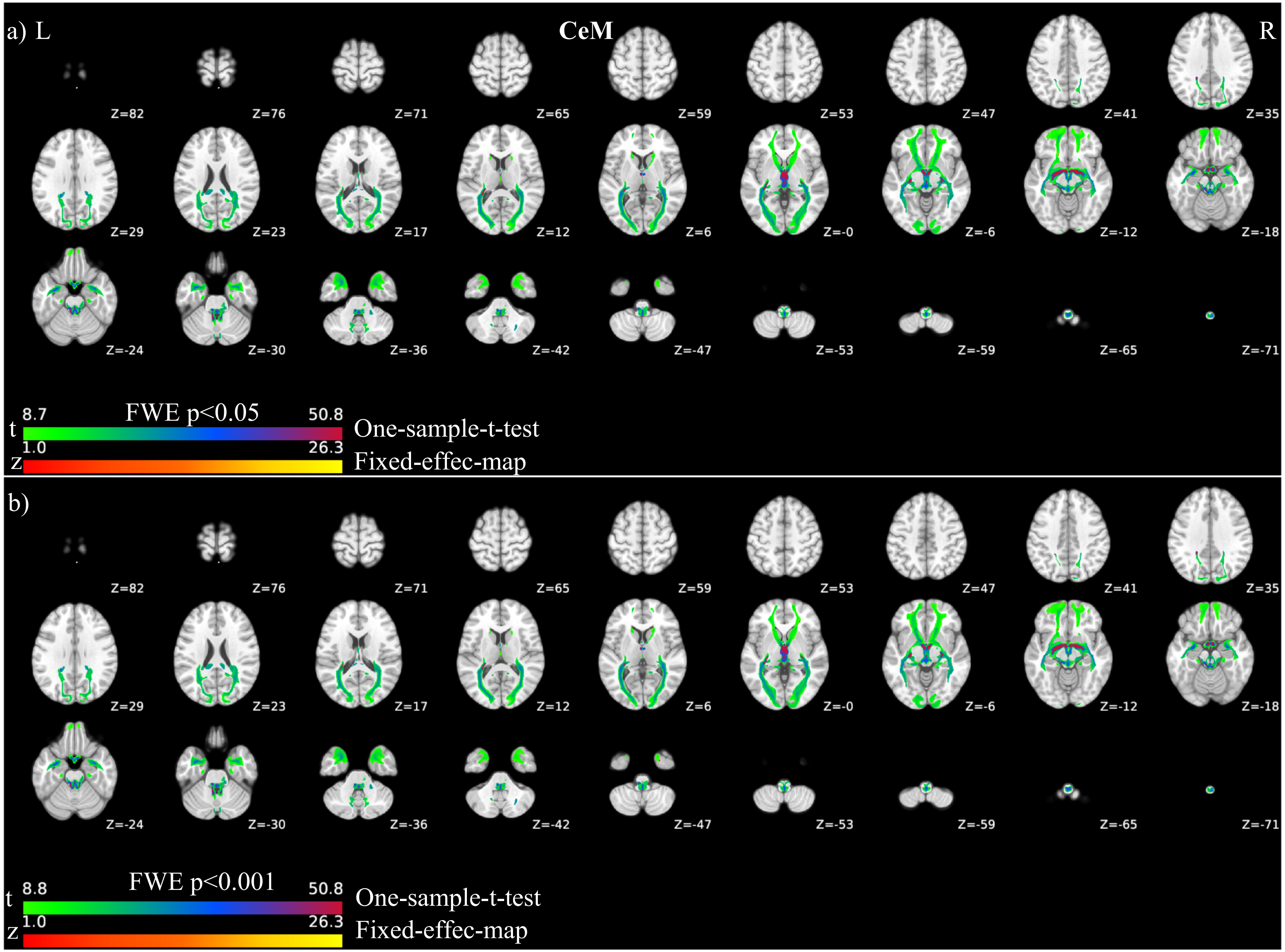

Supplementary Figure 8: Central medial nucleus (CeM) - FWE corrected maps overlaid on Fixed-effect map: The one-sample t-test for left and right tracts was performed using SPM12. The FWE corrected, i.e., a) $p<0.05$ and b) $p<0.001$ maps, were masked with the fixed-effect maps (thr 1). GLM results are shown in the RBGYR20 Color code as an overlay, and FE in the PSYCH-FIXED color code as an underlay (provided in the Connectome workbench). Where the red to color is not visible indicates that the GLM results overlap entirely with the fixed-effect-maps.

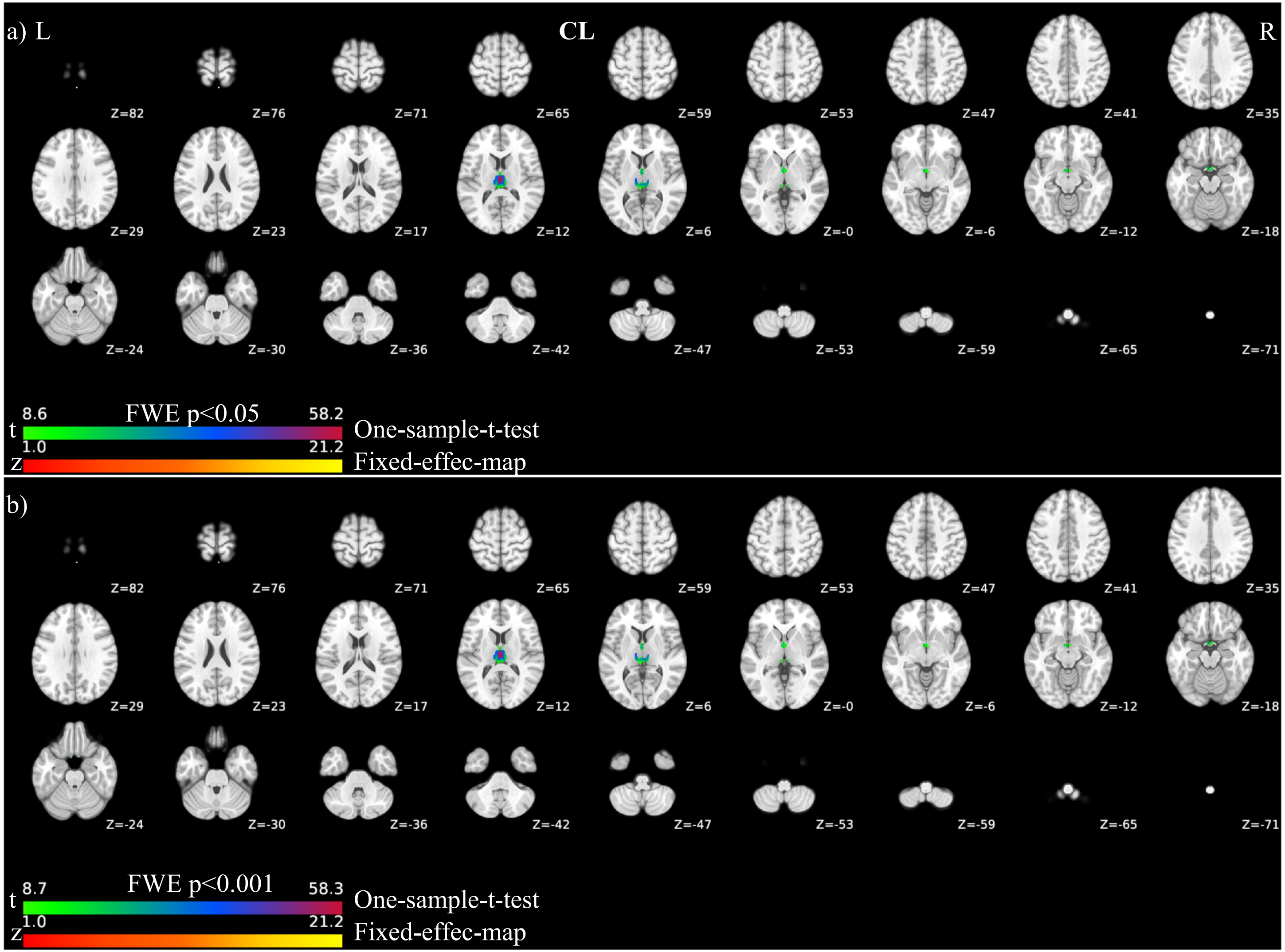

Supplementary Figure 9: Central lateral nucleus (CL) - FWE corrected maps overlaid on Fixed-effect map: The one-sample t-test for left and right tracts was performed using SPM12. The FWE corrected, i.e., a) $p<0.05$ and b) $p<0.001$ maps, were masked with the fixed-effect maps (thr 1). GLM results are shown in the RBGYR20 Color code as an overlay, and FE in the PSYCH-FIXED color code as an underlay (provided in the Connectome workbench). Where the red to color is not visible indicates that the GLM results overlap entirely with the fixed-effect-maps.

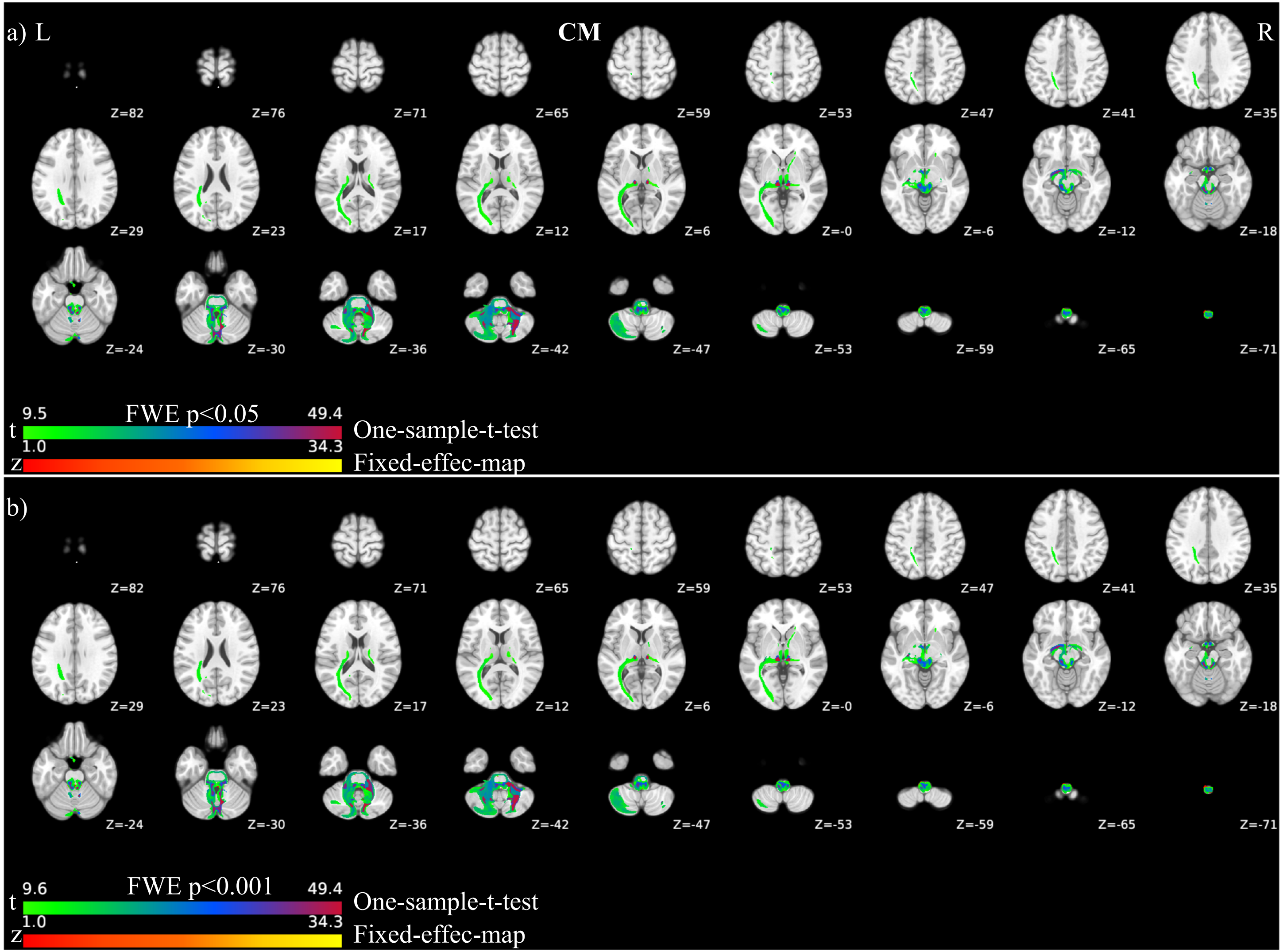

Supplementary Figure 10: Centromedian nucleus (CM) - FWE corrected maps overlaid on Fixed-effect map: The one-sample t-test for left and right tracts was performed using SPM12. The FWE corrected, i.e., a) $p<0.05$ and b) $p<0.001$ maps, were masked with the fixed-effect maps (thr 1). GLM results are shown in the RBGYR20 Color code as an overlay, and FE in the PSYCH-FIXED color code as an underlay (provided in the Connectome workbench). Where the red to color is not visible indicates that the GLM results entirely overlap with the fixed-effect-maps.

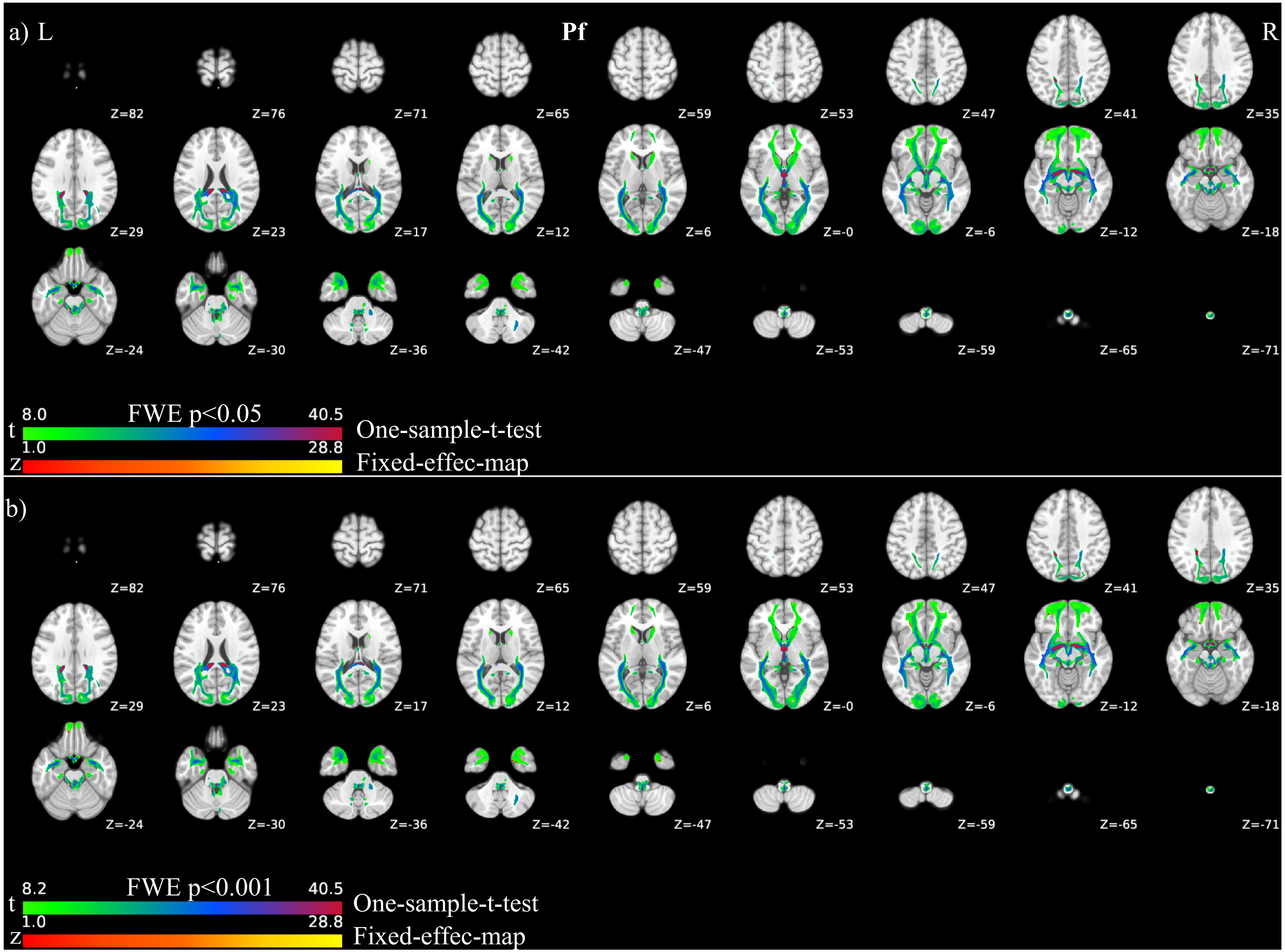

Supplementary Figure 11: Parafascicular nucleus (Pf) - FWE corrected maps overlaid on Fixed-effect map: The one-sample t-test for left and right tracts was performed using SPM12. The FWE corrected, i.e., a) $p<0.05$ and b) $p<0.001$ maps, were masked with the fixed-effect maps (thr 1). GLM results are shown in the RBGYR20 Color code as an overlay, and FE in the PSYCH-FIXED color code as an underlay (provided in the Connectome workbench). Where the red to color is not visible indicates that the GLM results entirely overlap with the fixed-effect-maps.

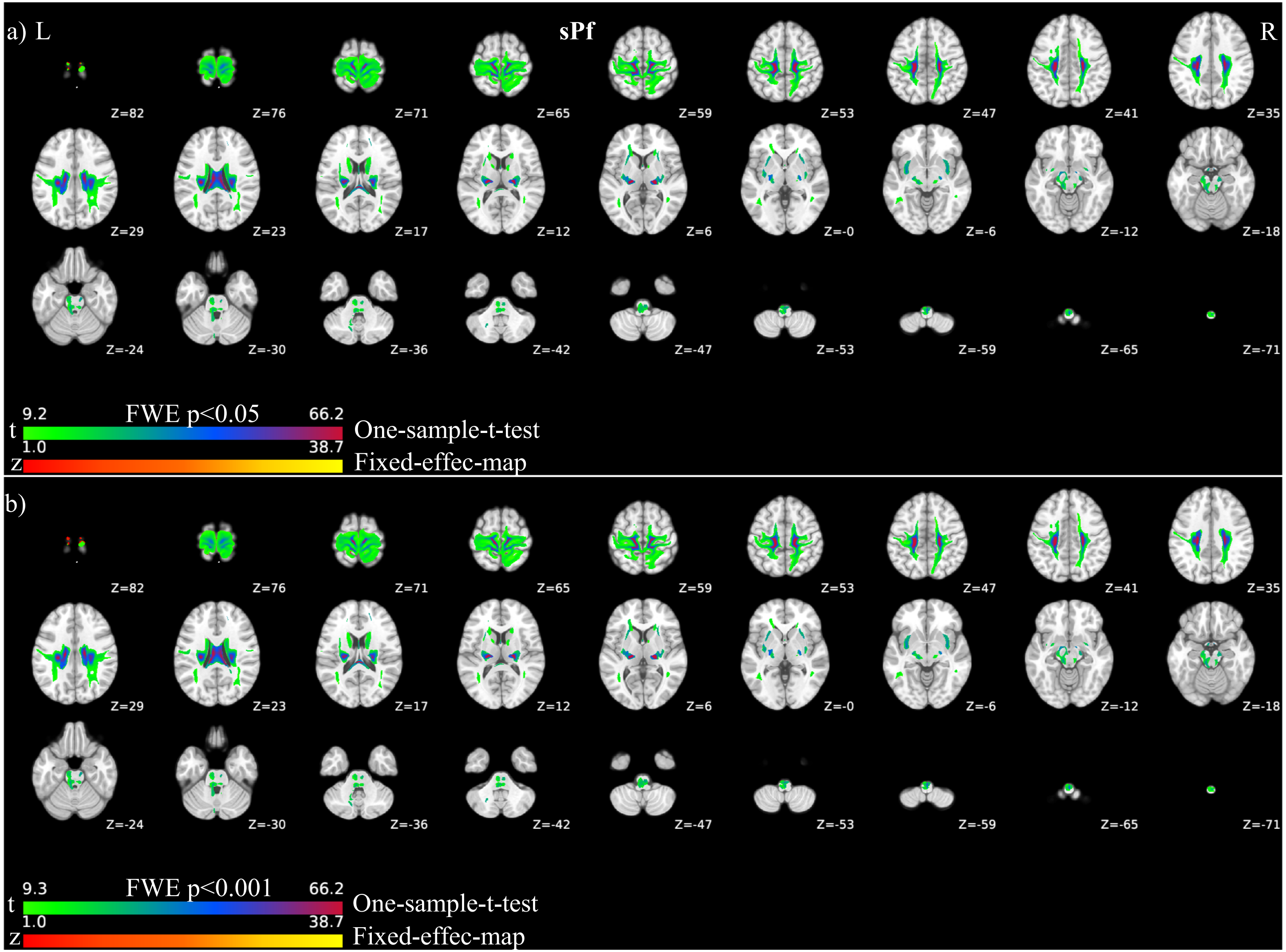

Supplementary Figure 12: Subparafascicular nucleus (sPf) - FWE corrected maps overlaid on Fixed-effect map: The one-sample t-test for left and right tracts was performed using SPM12. The FWE corrected, i.e., a) $p<0.05$ and b) $p<0.001$ maps, were masked with the fixed-effect maps (thr 1). GLM results are shown in the RBGYR20 Color code as an overlay, and FE in the PSYCH-FIXED color code as an underlay (provided in the Connectome workbench). Where the red to color is not visible indicates that the GLM results overlap entirely with the fixed-effect-maps.

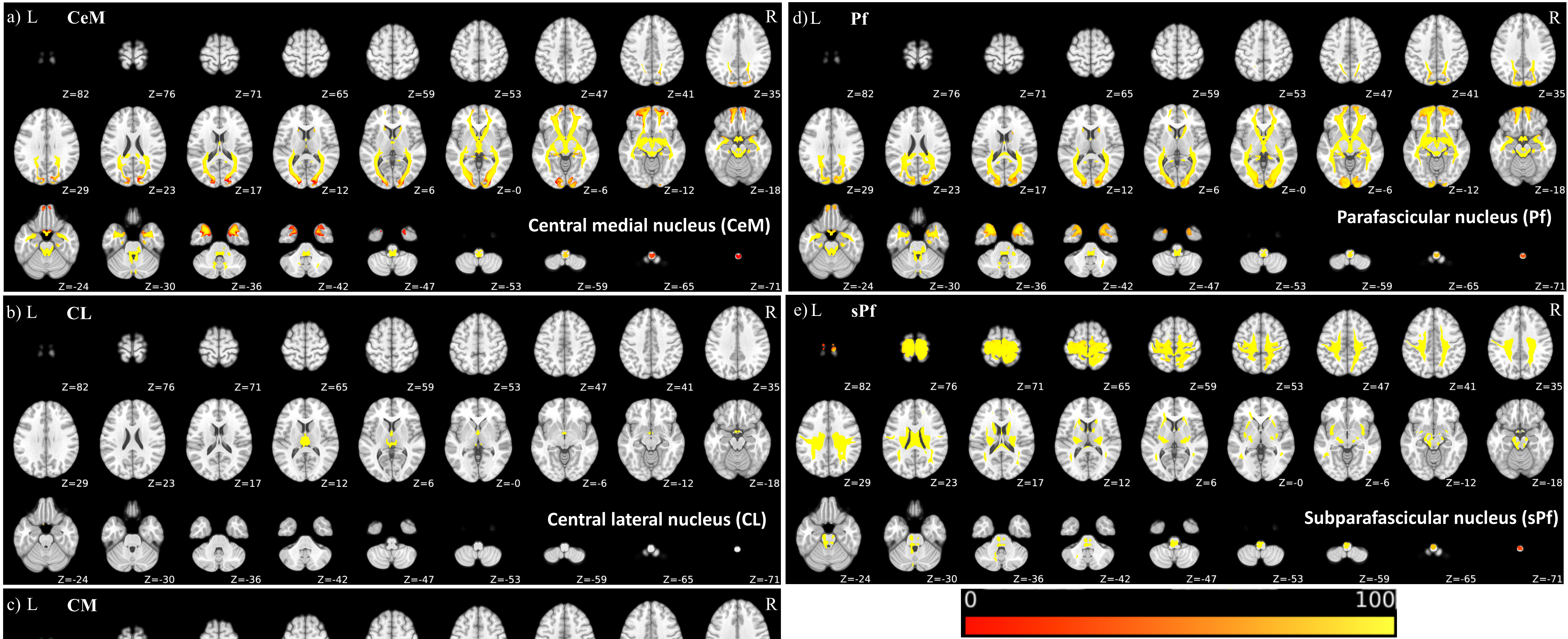

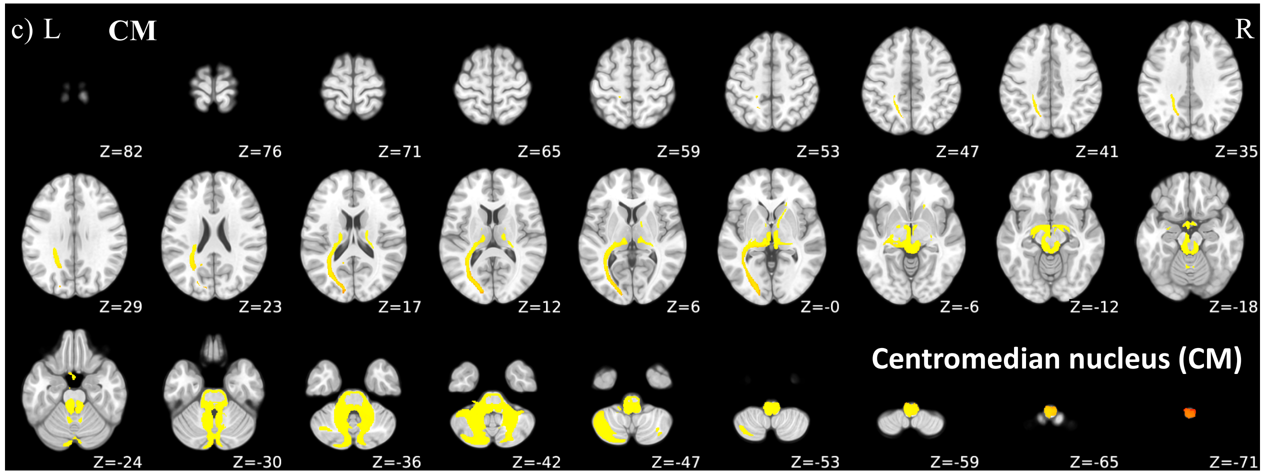

Supplementary Figure 13: Probability maps within the fixed-effect maps (thr 1): The red to yellow color code shows the increasing intensity of the probability map. The prominent yellow color indicates the high probability values in all maps, depicting the consistent connectivity patterns across the subjects for each Intralaminar nuclei. **Probability map calculation:** each subject‘s maps were binarized, and in the next step, all binarized maps were added and divided with the total number of subjects and multiplied by 100 to get the probability map.

Supplementary Text:
Methods:

Thalamus nuclei mask and Native space transformation:
(Source: https://fsl.fmrib.ox.ac.uk/fsl/fslwiki/FNIRT/UserGuide).

Linear Registration of B0 to T1 volume:
flirt -ref T1.nii.gz -in nodif_brain.nii.gz -dof 6 -omat nodif2struct.mat -out nodif2struct.nii.gz

Linear Registration of T1 to MNI:
flirt -ref MNI152_T1_2mm_brain -in T1.nii.gz -omat MNI2struct.mat -out MNI2struct.nii.gz

Non-Linear Registration of T1 to MNI:
fnirt --in=T1.nii.gz --aff=MNI2struct.mat --cout=my_nonlinear_transf --config=T1_2_MNI152_2mm

Inverse the transforms: MNI to Diffusion-native-space:

"inverse non-linear transform"
invwarp --ref=T1.nii.gz --warp=my_nonlinear_transf --out=warps_into_my_struct_space

"Inverse nodif2struct.mat"
convert_xfm -omat inverse_nodif2struct.mat -inverse nodif2struct.mat

Register MNI spaced ROIs to Subject-space using Linear and Non-linear registration:
applywarp --ref=nodif_brain.nii.gz --in=MNI_spaced_ROI --warp=warps_into_my_struct_space --postmat=inverse_nodif2struct.mat --out=ROI_diff_space --interp=nn

Native-subject-space Tractogram registration to MNI Space:
(Source: https://fsl.fmrib.ox.ac.uk/fsl/fslwiki/FNIRT/UserGuide).

Linear Registration of B0 to T1 volume:
flirt -ref T1.nii.gz -in nodif_brain.nii.gz -dof 6 -omat nodif2struct.mat -out nodif2struct.nii.gz

Linear Registration of T1 to MNI:
flirt -ref MNI152_T1_1mm_brain -in T1.nii.gz -omat MNI1struct.mat -out MNI1struct.nii.gz

Non-Linear Registration of T1 to MNI:
fnirt --in=T1.nii.gz --aff=MNI1struct.mat --cout=MNI1mm_NL_transf --config=T1_2_MNI152_1mm

Register Tractograms to MNI space using Linear and Non-linear registration:
applywarp --ref=MNI152_T1_1mm --in=tract.nii.gz --warp=MNI1mm_NL_transf --premat=nodif2struct.mat --out=my_warped_nodif_1mm

CeM and Pf Connectivity Comparison:

The CeM and Pf connectivity patterns (Figure 5-6, Supplementary Figure 3-4) almost resemble each other. The detailed analysis of the differences between the CeM and Pf revealed a slightly more pronounced Visual Cortex V1 in the right Pf, in contrast to the CeM (Supplementary Table 11). In contrast, the Superior parietal lobule 7P is slightly pronounced in the right CeM, unlike the Pf (Supplementary Table 11). The cerebellar lobule-specific assignments, i.e., lobule I-IV, Crus II, and Right IX, are slightly pronounced in the right CeM compared to the Pf (Supplementary Table 8). The brainstem nuclei-specific assignments show slightly higher overlap with CeM, in contrast to the Pf connectivity map (Supplementary Table 7).
